# Structural basis of redox modulation on chloroplast ATP synthase

**DOI:** 10.1038/s42003-020-01221-8

**Published:** 2020-09-02

**Authors:** Jay-How Yang, Dewight Williams, Eaazhisai Kandiah, Petra Fromme, Po-Lin Chiu

**Affiliations:** 1 Center for Applied Structural Discovery (CASD), Biodesign Institute, Arizona State University, Tempe, AZ USA; 2 Eyring Materials Center, Arizona State University, Tempe, AZ 85287 USA; 3 European Synchrotron Radiation Facility, 38000 Grenoble, France; 4 School of Molecular Sciences, Arizona State University, Tempe, AZ 85287 USA

**Keywords:** Bioenergetics, Cryoelectron microscopy

## Abstract

In higher plants, chloroplast ATP synthase has a unique redox switch on its γ subunit that modulates enzyme activity to limit ATP hydrolysis at night. To understand the molecular details of the redox modulation, we used single-particle cryo-EM to determine the structures of spinach chloroplast ATP synthase in both reduced and oxidized states. The disulfide linkage of the oxidized γ subunit introduces a torsional constraint to stabilize the two β hairpin structures. Once reduced, free cysteines alleviate this constraint, resulting in a concerted motion of the enzyme complex and a smooth transition between rotary states to facilitate the ATP synthesis. We added an uncompetitive inhibitor, tentoxin, in the reduced sample to limit the flexibility of the enzyme and obtained high-resolution details. Our cryo-EM structures provide mechanistic insight into the redox modulation of the energy regulation activity of chloroplast ATP synthase.

## Abstract (web summary)

Jay-How Yang et al. use single-particle cryo-EM to determine the structures of spinach chloroplast ATP synthase in reduced and oxidized states. They report a torsional constraint in the oxidized γ subunit that is alleviated by free cysteines in the reduced state. Their work provides mechanistic insights into the redox modulation of the ATP synthesis by the chloroplast ATP synthase.

## Introduction

ATP synthase is a molecular motor that converts energy from a membrane electrochemical potential into the high energy phosphate bonds in the ATP molecule, which is utilized throughout the cell to sustain its life by hydrolyzing ATP to ADP. ATP synthase is present in all forms of life, ranging from bacteria to animals and plants^1,2^. In a healthy living cell, mitochondrial and bacterial membranes are always energized for maintaining the routine activities of the cell, whereas photosynthetic membranes are de-energized during the night when no light energy is available to facilitate energy production via the photosynthetic electron transport chain. The ATP synthase motor can rotate reversibly in the opposite direction, if there is no regulatory control, to consume ATP molecules that are generated during the day^3^. Thus, to minimize energy waste, photosynthetic organisms have developed a fast, light-dependent mechanism to prevent the ATP synthase from hydrolyzing ATP molecules at night^4,5^.

Chloroplast ATP synthase (CF_1_F_O_) from higher plants has a unique redox switch that serves to modulate the ATP synthesis activity^4,6,7^. The CF_1_F_O_ enzyme actively synthesizes ATP in the reduced state, whereas the oxidized form has a low activity^6,8^. At sunrise, in the thylakoid membrane, the photosynthetic electron transport chain, consisting of photosystem II, cytochrome *b*6*f* complex, and photosystem I, performs a light-induced charge separation, which energizes the membrane and creates an electrochemical gradient^9^. This gradient activates the CF_1_F_O_, which releases a tightly bound ATP and enters an active but still oxidized state, synthesizing ATP molecules at a slower rate^10–12^. Meanwhile, PSI changes the redox state of the chloroplasts by reducing ferredoxin protein, which docks to the ferredoxin NADP reductase and enables the reduction of NADP^+^ to NADPH. Ferredoxin also acts as a reductant messenger to reduce thioredoxin, which in turn reduces and activates the CF_1_F_O_^13,14^, leading to the synthesis of ATP at a full rate of more than 200 ATP molecules per second^4^. At night, the plant needs to prevent the enzyme from wasting energy in the absence of light, since the CF_1_F_O_ can also hydrolyze ATP by rotating in the opposite direction^15^. A general hypothesis is that the oxidization of the CF_1_F_O_ limits this rotary action, thereby hindering unnecessary ATP hydrolysis^13,14^.

In recent years, single-particle electron cryogenic microscopy (cryo-EM) has become a powerful tool in the study of ATP synthase structures^16^. In vitro biochemical assays and mutagenesis have shown that the redox state modulates the CF_1_F_O_ activities^4–6,8,17^, and cryo-EM imaging of the CF_1_F_O_ in an autoinhibited and oxidized state has shown a unique disulfide linkage in the γ subunit (γCys240–γCys246)^18^, which stabilizes the local structure of the two β hairpin motifs of the γ subunit. Although the cryo-EM structure of the oxidized CF_1_F_O_ has been generated, structural information of the reduced form is still missing. To understand the molecular mechanism of the redox modulation of CF_1_F_O_, a more complete structural view is required in order to provide a fundamental framework of energy regulation in plants.

To investigate the structure of CF_1_F_O_ in different redox states, we isolated and purified the full enzyme complex from spinach leaves (*Spinacia oleracea*). We modulated the redox state of the enzyme using external redox agents, dithiothreitol (DTT) and iodosobenzoate (IBZ), and characterized the activities of ATP synthesis^19–23^. Single-particle cryo-EM was utilized to determine the CF_1_F_O_ structures, with a total of nine full complex structures (including the control) at resolutions in the range of 3.4–7.9 Å. Among the individual redox states, the particle images could be categorized into three distinct rotary states. We further focused the density refinement on the F_1_ domains, which have improved map resolutions from 3.0 to 4.4 Å. These cryo-EM density maps allowed us to build atomic models of CF_1_F_O_ under the redox states in question, giving insights into the mechanisms of redox modulation on the enzyme activity.

## Results

### Characterization of the CF_1_F_O_ redox states

**Fig. 1:**
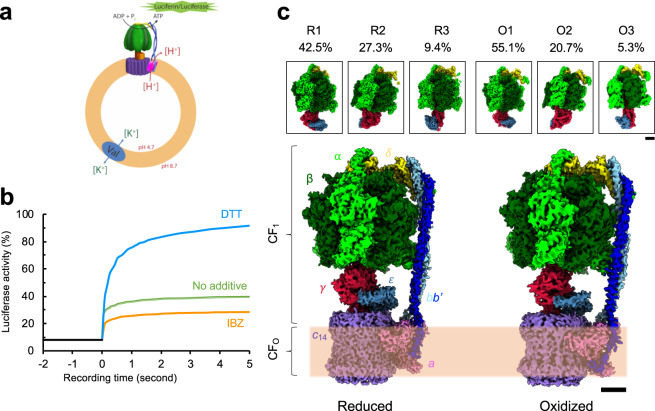
Chloroplast ATP synthase (CF_1_F_O_) of *Spinacia oleracea* in two different redox states. **a** Schematics of the experimental design for measuring CF_1_F_O_ function. Purified CF_1_F_O_ was reconstituted into a liposome (orange) mixed with lipids of phosphatidylcholine and phosphatidic acid. The generated pH gradient across the membrane drove the reconstituted CF_1_F_O_ to synthesize ATP molecules, which were detected using a luciferin/luciferase assay (green). Val indicates valinomycin. **b** Profile of the CF_1_F_O_ activity measurements of the CF_1_F_O_ in different redox states. Blue curve represents the sample with dithiothreitol (DTT) (reduced), orange the sample with iodosobenzoate (IBZ) (oxidized), and green the sample with no redox agent added (control). **c** Cryo-EM density maps of the oxidized and reduced forms of the CF_1_F_O_. Percentages of the particle images used are listed for individual rotary states. Scale bars indicate 25 nm. Color codes: α (light green), β (dark green), δ (yellow), *bb'* (blue and light blue), γ (crimson), ε (indigo), *a* (light pink), and *c* ring (purple). R indicates a reduced state, and O indicates an oxidized state. Membrane bilayer is indicated with the light orange band. The three-dimensional (3D) reconstructions are categorized into three different rotary states (states 1, 2, and 3). Upper insets are the density maps of the F_1_ domains.

It has been shown that the reduced form of the plant CF_1_F_O_ is more active in producing ATP molecules than the oxidized form, and the rate of the ATP synthesis of the reduced form is much faster than that of the oxidized form^4^. To produce the CF_1_F_O_ in the various redox states, we first isolated the full CF_1_F_O_ complex from spinach leaves (Supplementary Fig. 1a, b) and reconstituted it in the membrane bilayer of liposomes with a pH gradient established across the membrane using the ΔpH-step jump method. We then applied DTT or IBZ as the reducing and oxidizing agents, respectively, to mix with the reconstituted liposomes, generating different redox states of the CF_1_F_O_. One sample without DTT or IBZ treatment was taken as the control sample. ADP was supplied to initiate the ATP synthesis reaction, and the generated ATP molecules were detected using a luciferin–luciferase assay^19–23^ (Fig. 1a, b). Using the curve of the control sample as a reference, luciferase activity of the reduced sample was higher than that of the oxidized sample (Fig. 1b). This supports the conclusion that the CF_1_F_O_ redox state can be modulated using these external redox agents, thus changing the enzymatic activity accordingly.

### Single-particle cryo-EM of the CF_1_F_O_

Different redox states were generated by the external reagents and imaged by cryo-EM for three-dimensional (3D) reconstruction. As prepared for the functional measurements, three different protein samples in detergent micelles were imaged: reduced (DTT), oxidized (IBZ), and control enzyme complexes. The electron micrographs of individual groups showed a homogeneous distribution of the purified CF_1_F_O_ enzyme complex (Supplementary Figs. 2a–e, 3a–e, 4a–e). After iterative 3D classification procedures, the reconstructions of three different rotary states were categorized and determined in individual groups. Comparison of the three different redox states showed that rotary state 1 was the most populated, while rotary state 3 was the least populated (Supplementary Figs. 2b, 3b, 4b). The percentage of the particle images of the reduced enzyme consisting of rotary state 2 (27.3%) and 3 (9.44%) are higher than in the oxidized enzyme (rotary state 2: 20.7%; rotary state 3: 5.26%) (Supplementary Figs. 2b, 3b, 4b). In each sample, about ~20% of the total particle images could not be unambiguously categorized into any of the three rotary states; these uncategorized particle images were possibly the 2D projections with the particles in the intermediate rotary states or ones lacking discernible features. All the resulting 3D density maps unambiguously feature the structural elements of the extracellular F_1_ domain and the membrane F_O_ part. By increasing the map contours, the inside of the detergent-bound region can be distinguished from the extracellular domains, and the 14 hairpin structures of the *c*_14_ ring rotor were clearly resolved (Supplementary Figs. 2e, 3e, 4e). The resolutions among the density maps of the full complexes ranged from 3.4 to 7.9 Å (CF_1_ 3.0 to 4.4 Å) (Fig. 1c; Supplementary Figs. 2c, 3c, 4c, 5). Although the density maps generated from the control dataset could be categorized into three different rotary states (Supplementary Fig. 4), they were generated at relatively low resolutions (5.2–7.9 Å). One explanation could be that the control dataset was composed of multiple states, conferring a greater conformational variability in the population and thus flattening out the high-resolution details^24–26^. The statistics of data processing are shown in Tables 1, 2, 3.

**Table 1:** Statistics of the single-particle cryo-EM structure of the reduced chloroplast ATP synthase of *Spinacia oleracea*.

	Chloroplast ATP synthase of *Spinacia oleracea* in dithiothreitol (DTT)		
	R1	R2	R3
	(EMD-21270, EMD-21271)	(EMD-21268, EMD-21269)	(EMD-21266, EMD-21267)
	(PDB codes: 6VON, 6VOO)	(PDB codes: 6VOL, 6VOM)	(PDB codes: 6VOJ, 6VOK)
Data collection and processing			
Magnification		130,000×	
Voltage (kV)		300	
Electron exposure (e^−^/Å^2^)		49	
Defocus range (μm)		−1.0 to −2.8	
Pixel size (Å)		1.053	
Symmetry imposed		C1	
Initial particle images (no.)		108,691	
Final particle images (no.)	46,180	29,667	10,264
Map resolution (Å)	3.35 (3.05)	4.06 (3.60)	4.34 (3.85)
FSC threshold			
Refinement			
Initial model used (PDB code)	6FKF	6FKH	6FKI
Model resolution (Å)	3.3 (3.1)	3.9 (3.6)	4.2 (3.7)
FSC threshold of 0.143			
Cross correlation			
Masked	0.718 (0.760)	0.705 (0.702)	0.668 (0.708)
Volume	0.704 (0.747)	0.718 (0.702)	0.676 (0.713)
Map sharpening *b* factor (Å^2^)	−50.65 (−34.10)	−64.42 (−61.15)	−76.98 (−60.57)
Model composition			
Non-hydrogen atoms	39,163 (27,551)	38,991 (27,492)	39,155 (27,512)
Protein residues	5186 (3574)	5165 (3566)	5185 (3569)
Ligands			
ATP	4	4	4
ADP	2	2	2
Tentoxin	1	1	1
*B* factors (Å^2^)			
Protein	52.25 (58.02)	33.99 (45.03)	63.72 (40.88)
Ligands	26.06 (44.20)	22.88 (34.80)	30.79 (26.45)
RMS deviations			
Bond lengths (Å)	0.005 (0.006)	0.005 (0.005)	0.005 (0.007)
Bond angles (°)	0.861 (0.787)	0.848 (0.862)	0.899 (0.974)
Validation			
MolProbity score	1.85 (1.90)	1.98 (1.98)	1.92 (2.03)
Clashscore	8.30 (8.99)	12.48 (11.60)	9.88 (10.75)
Poor rotamers (%)	0.05 (0.03)	0.12 (0.10)	0.34 (0.51)
Ramachandran plot			
Favored (%)	94.08 (93.64)	94.66 (93.88)	94.04 (92.31)
Allowed (%)	5.82 (6.21)	5.20 (5.95)	5.88 (7.58)
Disallowed (%)	0.10 (0.14)	0.14 (0.17)	0.08 (0.11)

Values in the parentheses are the refinement statistics for the F_1_ region.

**Table 2:** Statistics of the single-particle cryo-EM structure of the oxidized chloroplast ATP synthase of *Spinacia oleracea*.

	Chloroplast ATP synthase of *Spinacia oleracea* in iodosobenzoate (IBZ)		
	O1	O2	O3
	(EMD-21264, EMD-21265)	(EMD-21262, EMD-21263)	(EMD-21241)
	(PDB codes: 6VOH, 6VOI)	(PDB codes: 6VOF, 6VOG)	(PDB codes: 6VMG)
Data collection and processing			
Magnification		48,077×	
Voltage (kV)		300	
Electron exposure (e^−^/Å^2^)		44.4	
Defocus range (μm)		−1.5 to −4.0	
Pixel size (Å)		1.04	
Symmetry imposed		C1	
Initial particle images (no.)		552,893	
Final particle images (no.)	304,879	114,542	29,090
Map resolution (Å)	4.16 (4.03)	4.51 (4.35)	6.46
FSC threshold			
Refinement			
Initial model used (PDB code)	6FKF	6FKH	6FKI
Model resolution (Å)	4.1 (3.9)	4.4 (4.3)	6.6
FSC threshold of 0.143			
Cross correlation			
Masked	0.728 (0.752)	0.774 (0.824)	0.711
Volume	0.712 (0.734)	0.773 (0.821)	0.696
Map sharpening *b* factor (Å^2^)	−211.95 (−203.09)	−179.39 (−232.12)	−304.57
Model composition			
Non-hydrogen atoms	39,159 (27,497)	35,182 (27,137)	25,375
Protein residues	5193 (3575)	5175 (3563)	5170
Ligands			
ATP	4	4	0
ADP	1	1	0
*B* factors (Å^2^)			
Protein	84.67 (64.35)	144.23 (158.45)	222.15
Ligands	68.15 (55.87)	119.07 (142.64)	
RMS deviations			
Bond lengths (Å)	0.005 (0.005)	0.007 (0.006)	0.006
Bond angles (°)	0.941 (0.881)	0.983 (1.025)	0.970
Validation			
MolProbity score	2.14 (2.12)	2.19 (2.20)	2.05
Clashscore	14.08 (13.35)	15.13 (15.24)	9.65
Poor rotamers (%)	0.49 (0.24)	0.76 (0.49)	0.00
Ramachandran plot			
Favored (%)	92.02 (91.96)	91.63 (91.11)	90.31
Allowed (%)	7.68 (7.73)	8.32 (8.80)	9.60
Disallowed (%)	0.29 (0.31)	0.06 (0.08)	0.10

Values in the parentheses are the refinement statistics for the F_1_ region.

**Table 3:** Statistics of the single-particle cryo-EM structure of the control chloroplast ATP synthase of *Spinacia oleracea*.

	Chloroplast ATP synthase of *Spinacia oleracea*		
	C1	C2	C3
	(EMD-21239, EMD-21240)	(EMD-21238)	(EMD-21235)
	(PDB codes: 6VMB, 6VMD)	(PDB codes: 6VM4)	(PDB codes: 6VM1)
Data collection and processing			
Magnification		48,077×	
Voltage (kV)		300	
Electron exposure (e^−^/Å^2^)		43.5	
Defocus range (μm)		−1.5 to −4.0	
Pixel size (Å)		1.04	
Symmetry imposed		C1	
Initial particle images (no.)		208,371	
Final particle images (no.)	127,760	26,291	13,947
Map resolution (Å)	5.23 (4.53)	7.08	7.90
FSC threshold			
Refinement			
Initial model used (PDB code)	6FKF	6FKH	6FKI
Model resolution (Å)	6.5 (4.5)	7.2	7.9
FSC threshold of 0.143			
Cross correlation			
Masked	0.764 (0.841)	0.787	0.786
Volume	0.753 (0.836)	0.764	0.768
Map sharpening *b* factor (Å^2^)	−187.09 (−195.21)	−269.31	−507.69
Model composition			
Non-hydrogen atoms	35,401 (27,501)	25,445	25,331
Protein residues	5194 (3576)	5184	5161
Ligands			
ATP	4	0	0
ADP	1	0	0
*B* factors (Å^2^)			
Protein	306.88 (130.00)	376.31	286.00
Ligands	233.70 (101.89)		
RMS deviations			
Bond lengths (Å)	0.006 (0.008)	0.007	0.007
Bond angles (°)	1.064 (1.097)	0.948	1.054
Validation			
MolProbity score	2.01 (2.18)	1.98	2.27
Clashscore	10.54 (12.90)	7.94	12.03
Poor rotamers (%)	0.51 (0.82)	0.00	0.00
Ramachandran plot			
Favored (%)	92.59 (89.74)	90.02	84.46
Allowed (%)	7.23 (10.12)	9.90	15.42
Disallowed (%)	0.18 (0.14)	0.08	0.12

Values in the parentheses are the refinement statistics for the F_1_ region.

Atomic models that were built along with the resulting cryo-EM density maps (Supplementary Figs. 6, 7, 8) showed that the overall architectures of the three different rotary states in both redox groups. The F_1_ domain is composed of a catalytic α_3_β_3_ hexamer, a δ stator, a γ-ε central shaft, and the extracellular domain of a heterodimeric *bb*′ stator. Our structure of the oxidized state is in good agreement with previous structures of the oxidized state of the CF_1_F_O_, while significant differences are revealed when the structure of the inactive oxidized state was compared to our first revealed structure of the active, reduced state of the CF_1_F_O_. The packing of the catalytic unit with the δ and *bb*′ stators is the same as in both oxidized and reduced forms (Supplementary Fig. 9a, b). The interactions between the catalytic head and the peripheral stalk are essential to stabilize the F_1_ domain during the process of ATP synthesis occurring at the αβ interfaces and triggered by the rotation of the central shaft via the binding change mechanism^27,28^.

### Nucleotide-binding states in α_3_β_3_ catalytic unit

According to the binding change mechanism, the asymmetric α_3_β_3_ hexamer with the γ-ε central shaft alternates the three nucleotide-binding sites on the αβ interfaces for ATP synthesis^27,29^. These three sites are identified as loose (partially open, ADP and phosphate bound, β_L_), tight (closed, ATP bound, β_T_), and open (empty, β_O_) sites^27^. Note that the γ-ε central shaft faces the β_O_ open site^30^. In our cryo-EM densities of both the reduced and oxidized forms, three ATP molecules were modeled into the clearly defined densities in the α subunits (Supplementary Fig. 10a, b). Our cryo-EM density maps allow us to identify one ADP located in the loose β_L_ catalytic site and one ATP located in the tight β_T_ site (Supplementary Fig. 10a). This finding fully agrees with the binding change mechanism proposed by Boyer^27^. The energy generated from the proton gradient then changes the binding affinity and conformation of the F_1_ domain, leading to the release of ATP molecule. We were now for the first time able to identify an ATP in the tight binding site of the CF_1_F_O_, in contrast to the previous structures of the inactive, oxidized chloroplast ATP synthase (PDB codes: 6FKF, 6FKH, and 6FKI), which showed the ADPs occupying both the β_L_ and β_T_ sites^18^. We show the interactions of the bound nucleotides in these binding sites are conserved: the aromatic side chains of the βTyr362 and βPhe441 stack with the adenine of the nucleotide and βLys178, βThr179, and αArg366 (arginine finger) interact with the phosphate group (Supplementary Fig. 10a, b). The β_O_ open site does not have any density for a nucleotide. Thus, the locations of the nucleotide binding in our structures are consistent with previous findings^28^. In contrast to Hahn et al.^18^, our oxidized CF_1_F_O_ structures match the nucleotide-binding occupancy proposed in the binding change mechanism^27^.

**Fig. 2:**
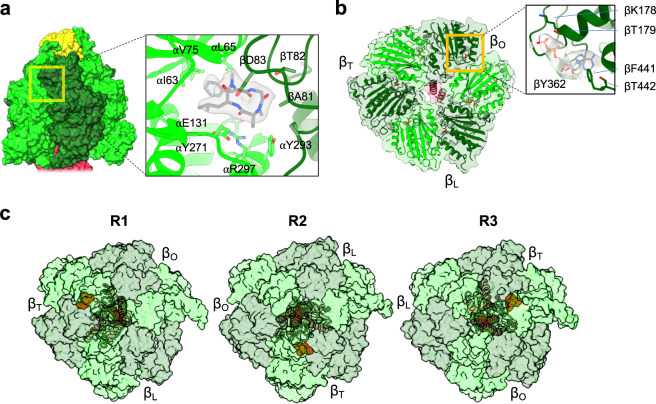
Structure of the reduced CF_1_ domain. **a** Tentoxin-binding site. Surface overview of the F_1_ domain is shown on the left, with an enlarged view of the tentoxin-binding site shown on the right. Light green, dark green, and yellow are the α, β, and δ subunits, respectively. Tentoxin and its cryo-EM density are shown in gray. **b** Cross section of the α_3_β_3_ catalytic unit from the reduced CF_1_F_O_. Central coiled coil is the central shaft γ subunit (crimson). ADP (gray stick) binds onto the β_O_ site in the enlarged view on the right. **c** Different rotary states bound with tentoxin. The tentoxin binds to the αβ interface, where the β subunit associates with a β_T_ nucleotide-binding site. The αβ hexamers (light and dark green) are shown in surface representation and the central γ subunit (crimson) is shown in cartoon representation. Tentoxin molecules are shown as orange balls.

We had initially pursued single-particle cryo-EM of the reduced CF_1_F_O_ with the reducing agent DTT, but the resolution of the reconstructions was not high enough for us to model the atomic coordinates. This may be a result of the enzyme being highly flexible in its active, reduced form, leading to a low-resolution density map. To test this idea, we aimed to fix the CF_1_F_O_ in its active, reduced state in the native membrane by adding tentoxin after the enzyme was reduced before the enzyme was extracted from the membrane. The goal was to block the enzyme “in action” by restriction of the rotary action thereby limiting the mobility of the complex. Tentoxin, a cyclic peptide produced from the fungus *Alternaria* fungi, was reported to influence the CF_1_F_O_ activity in a concentration-dependent manner^31–33^. It blocks rotation and thereby multisite catalysis, but still allows for activation of single site catalysis^34^. Although the crystal structure of the F_1_ head complexed with tentoxin were determined previously, our cryo-EM structure is to our knowledge the first structure showing the intact enzyme in complex with tentoxin^33,35^. Our resulting cryo-EM densities imaged after the addition of tentoxin were overall well-resolved, corroborating the highly mobile nature of the reduced CF_1_F_O_. Our density maps also showed one consistent tentoxin-binding site, at the same site identified in previous crystal structure (PDB code: 1KMH)^33^ (Fig. 2a). Tentoxin binds to the interface between the α and β subunit (with the β_T_ binding site). Its cyclic ring interacts with the charged or polar residues (βAsp83, βThr82, αGlu131, αArg297, and αTyr271), and its isobutyl and phenyl moieties interact with the hydrophobic residues (αIle63, αLeu65, and αVal75) (Fig. 2a, c). Our structure of the reduced CF_1_F_O_ reveals that the tentoxin binds specifically to the α-β_T_ interface thereby blocking the most critical and rate limiting step in the rotary mechanism: the opening of the β_T_ site and the release of the ATP molecule. All three rotary states of the active, reduced CF_1_F_O_ showed the density of the bound tentoxin ligand, and its binding site alternates with the rotary state (Fig. 2c). Thus, in the three rotary states, the tentoxin-binding site is alternated together with the β_T_ subunit, which indicates that tentoxin-binding indeed fixed each of the rotary state of the active, reduced enzyme.

In addition to the identification of the tentoxin-binding site, the density of one additional ADP molecule was found in the β open site (β_O_) (Fig. 2b), which did not appear in the oxidized or control structures, nor in the previous structure of the oxidized CF_1_F_O_^18^. The density of this extra ADP molecule was clearly resolved, but we did not identify any additional density for the phosphate (Fig. 2b). This ADP is bound to the β subunit via interactions with the residues of βLys178, βThr179, βTyr362, and βPhe441 (Fig. 2b). Because the β_O_ binding site is away from the α subunit, the ADP has no interaction with the arginine finger (αArg366) of the adjacent α subunit. According to the binding change mechanism^27^, the β_O_ open site is both the entry site for the ADP and phosphate and the exit site for the ATP during the process of ATP synthesis^27^. It has also been shown that tentoxin inhibits the step of the ADP release from the β_O_ open site in ATP hydrolysis^32,36^. Because ATP synthesis and hydrolysis are reversible processes for CF_1_F_O_, our tentoxin-bound and reduced CF_1_F_O_ structure is to our knowledge very likely the first visualization of the entry step of the ADP molecule for the subsequent ATP synthesis.

### γ-ε central shaft and the redox switch

**Fig. 3:**
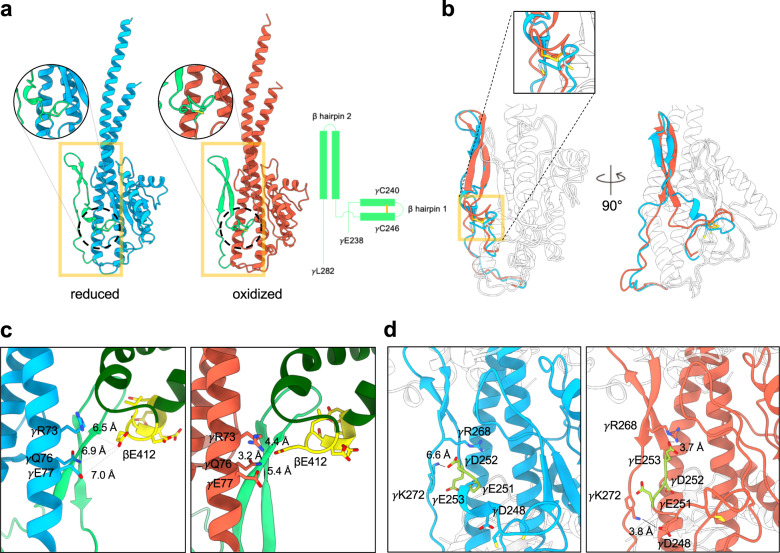
Structures of the reduced and oxidized γ subunits. **a** Structures of the reduced (light blue) and oxidized (orange) γ subunits. Two β hairpin structures (from γGlu238 to γLeu282) are shown in light green, and the two cysteines of the redox switch are shown in yellow in circular enlarged views. Diagram on right shows the topology of the two β hairpin structures. **b** Superposition of the reduced and oxidized γ subunits (RMSD 1.016 Å). The two β hairpins are shown in light blue and orange for the reduced and oxidized forms, respectively. Other regions are shown in white. **c** Interaction networks of the β hairpin 2 and βDELSEED motif. Left and right panels are the reduced (γ subunit in light blue) and oxidized (γ subunit in orange) forms. Light green represents the β hairpin 2, dark green for the β subunit, and yellow for the βDELSEED motif. The distances connecting the residues of the γ coiled coil (γArg73, γGln76, and γGlu77) with the βGlu412 are labeled. **d** Interaction of the EDE motif with the γ subunit. The EDE motif (yellow) does not interact with any part of the reduced γ subunit but forms an extensive interaction network with its neighborhood when the γ subunit is oxidized.

The two cysteines, γCys240 and γCys246, comprise the redox switch in the γ subunit of the CF_1_F_O_^4,18^. They are located on a short β hairpin loop motif (β hairpin 1), connected to a long anti-parallel β hairpin motif (β hairpin 2) at its C-terminus (Fig. 3a, b). Our oxidized γ subunits are similar to the previous structures (PDB code: 6FKF; RMSD of the γ subunits: 0.877 Å)^18^. Superposition of the reduced and oxidized γ subunits showed the same architecture of the secondary structures (RMSD 1.016 Å), but the local structures of the β hairpins 1 and 2 are different (γGlu238 – γLeu282) (Fig. 3b). The oxidized γ subunit structure shows that the β hairpin 1 is formed by the disulfide bond formation and the β hairpin 2 is stabilized by two long anti-parallel β strands with extensive hydrogen bond pairs between their N–H and C = O groups on the peptide bonds (Fig. 3a, b). However, the reduced γ subunit does not maintain the β hairpin 1 structure due to the disconnection of the disulfide bond, and the β strands of the β hairpin 2 are shorter than those of the oxidized form. The shorter β strands have less hydrogen bonding between the two long loops to stabilize the hairpin structure, and as such the disconnected cysteines are more likely to destabilize the structures of the two β hairpins. The reduced cysteines may release the torsional constraint on the β hairpin 1, forming a one-turn helix, uncoupling the β strands of the β hairpin 2, and destabilizing the loop structures of the γ subunit (Fig. 3a, b).

One motif with a helix-turn-helix structure in the β subunit has a highly conserved DELSEED sequence (βDELSEED motif) and features multiple negatively charged residues^37^. The conformation of the βDELSEED motif correlates with the nucleotide-binding state of the β subunit^37^ (Supplementary Fig. 11a). Generally, the interactions between the βDELSEED and the β hairpin 2 motifs do not seem to have a significant change in the two redox states (Supplementary Fig. 11a). Of the three reduced rotary states, the β hairpin 2 loop in the γ subunit has extensive interactions with the βDELSEED motif on the β subunit with the β_O_ site mainly via polar-polar (βSer414, βGlu416, βAsp417, γThr258, γThr259, γLys260, and γGlu267) and hydrophobic (βLeu408, βLeu413, γLeu257, and γLeu264) interactions (Fig. 3c; Supplementary Fig. 11b). Secondly, within the γ subunit, the aromatic side chain of the γPhe255 stacks with γPhe217 and buries within hydrophobic residues in the coiled coil of the γ subunit (γVal72, γVal79, γAla313, and γAla317)^18^. These interactions also contribute to stabilizing the interaction between the loop and βDELSEED motif, which are similar to the corresponding interactions in the oxidized state (Fig. 3c**;** Supplementary Fig. 11b).

The βGlu412 interacts with the γ central coiled coil in the oxidized state, but not in the reduced state (Fig. 3c). In the oxidized state, the side chain of the βGlu412 is very likely to form hydrogen bonds with the side chains of the γGln76 and γGlu77 (Fig. 3c). While in the reduced state, the βGlu412 is far from the γ coiled coil, which has less interaction with the central coiled coil and may result in less restriction for the central shaft rotation (Fig. 3c). On the other hand, the lower loop of the β hairpin 2 (γIle271 – γGlu285) has a low signal level (1.2 σ) in our cryo-EM density map of the reduced form, implying a high level of mobility. This entropic gain may also lead to less resistance for the central shaft rotation.

A previous mutagenesis study on the γ subunit showed that the mutant with the deletion of the three negatively charged residues, γGlu251, γAsp252, and γGlu253, was insensitive to redox regulation^38^. These three residues are located between the β hairpin 1 and 2, in the so-called EDE motif. In the reduced form, the EDE motif does not seem to interact with the adjacent residues, allowing the residues between γVal247 and γAla250 to form a short one-turn helix (Fig. 3d). In contrast, in the oxidized form, the EDE motif interacts with the adjacent residues and stabilizes the two β hairpin structures (Fig. 3d). Disulfide bond formation also introduces torsional stress to break the one-turn helix and form an anti-parallel β hairpin 2 (Fig. 3b). Thus, the oxidized form has an organized structure that provides a stable interaction network, and as a result, the oxidized γ subunit hampers the rotary action and has a slow rotation speed^4^.

The ε subunit binds peripherally and rotates together with the γ subunit. For bacterial F-type of ATP synthases, the ε subunit acts as a molecular switch to the catalysis by exerting a conformational transition of its C-terminal dual helices, which make up the so-called C-terminal domain (εCTD)^39–43^. However, a conformational change of the ε subunit was not observed in the CF_1_F_O_ structures. This may suggest a different role of the ε subunit in regulating CF_1_F_O_ given the low sequence similarity to the ε subunit of the *E. coli* and mitochondrial ATP synthases^44,45^.

### Membrane F_O_ domain

**Fig. 4:**
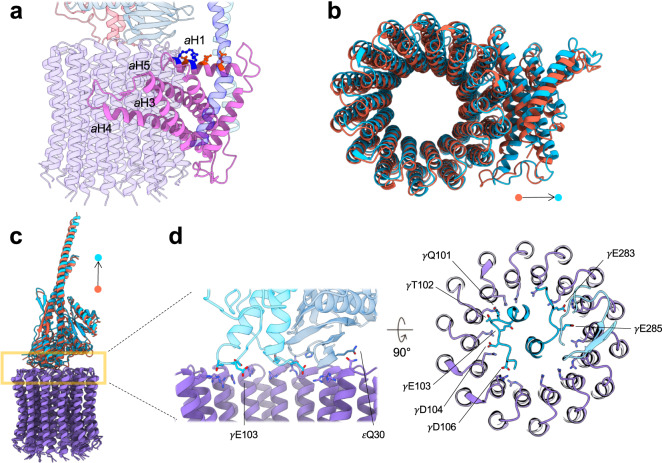
Interaction of the γ-ε central shaft with the membrane F_O_ domain. **a** Spatial arrangement of the subunit *a* in the membrane. Color codes: subunit *a* (light pink), c_14_ ring (purple), *bb*′ stator (blue and light blue), γ subunit (crimson), and ε subunit (steel blue). Charged residues on the *a*H1 are shown as a ball-and-stick model. Negatively (*a*Glu73, *a*Glu77, and *a*Asp81) and positively (*a*Arg80 and *a*Lys84) charged residues are shown in orange and blue, respectively. **b** Alignment of the membrane *c-*ring rotors of the reduced and oxidized states. Superposition shows the subunit *a* of the reduced CF_1_F_O_ is slightly away from the membrane *c* ring. **c** As in **b**, the superposition of the γ-ε central shafts of the reduced (light blue) and oxidized (orange) forms shows a slight translational movement of their centers of mass. **d** Interaction between the central shaft and the membrane *c* ring. Light blue and indigo represent the reduced γ subunit and ε subunit, respectively. *c*Arg41 are shown as sticks with their side chains mostly pointing to the ring center. In the γ and ε subunits, the negatively charged and polar residues that interact with the top of the *c* ring are shown as sticks.

The membrane F_O_ domain includes the *c*_14_ ring, subunit *a*, and the membrane part of the *bb*′ stator. Both the oxidized and reduced forms show the common overall F_O_ domain structure found in all F-type ATP synthase, which is consistent with the previously determined CF_1_F_O_ structure (PDB code: 6FKF, 6FKH, and 6FKI)^18^ (Fig. 1c; Supplementary Figs. 2, 3, 4). Subunit *a* is embedded in the membrane and sandwiched between the *bb*′ stator and *c* ring (Fig. 4a). The subunit *a* is composed of two half-channels responsible for proton translocation^46^, driving one full rotation of the *c*-ring rotor coupled to the synthesis of three ATP molecules^47^. The subunit *a* features a characteristic four-helix bundle from *a*H2 to *a*H5, binding peripherally to the *c* ring. The helix *a*H1 is parallel to the membrane plane with one side consisting of negatively charged residues (*a*Glu73, *a*Glu77, and *a*Asp81) facing the *c* ring and the peripheral *bb*′ stator, and the other side consisting of positively charged residues (*a*Arg80 and *a*Lys84) facing the membrane lipids (Fig. 4a). As the F_O_ domains of the reduced and oxidized structures are similar, we conclude that the redox modulation process does not affect the overall structure of the proton half-channels in the *a* subunit or the *c*_14_ membrane rotor ring. The previously reported crystallographic structures of the isolated *c*_14_ ring from the spinach CF_1_F_O_ showed a symmetric arrangement^48,49^. However, it is conceivable that the membrane ring may take on some level of flexibility to interact with the subunit *a* and the γ-ε central shaft. Our cryo-EM structures (Fig. 4d) showed that the residues on the top of the *c* ring are not symmetric, which may be caused by the electrostatic interactions between these conserved residues (*c*Arg41, *c*Gln42, and *c*Pro43) and the polar or acidic residues on the bottom of the γ-ε central shaft (γGln101, γThr102, γGlu103, γAsp104, γAsp106, γGlu283, γGlu285, and εGln30)^18,50,51^ (Fig. 4d).

To analyze the movements between the two redox states, we superimposed the membrane *c* ring and analyzed the spatial arrangements of other subunits in different redox and rotary states (Fig. 4c). Among the three rotary states, the membrane *c* rings of the reduced and oxidized forms were superimposed (Fig. 4b). An exciting finding is that the peripheral subunit *a* slightly moves away from the *c*_14_ ring when the enzyme is active, reduced state in all three rotary states (Fig. 4b and Supplementary Fig. 12). This implicates that the reduction of the γ subunit may have an influence in facilitating the rotation of the membrane *c*_14_ ring, thereby generating ATP molecules at a higher turnover speed (Fig. 1b).

The peripheral stators *bb*′ has a large tilt and shift in its ectodomain, and the α_3_β_3_ catalytic unit with the δ stator moves along with the *bb*′ stator (Supplementary Fig. 12). The tilting angles of the F_1_ catalytic domains around the axis perpendicular to the membrane plane are 1.74°, 5.89°, and 3.25° in the rotary state 1, 2, and 3, respectively (Supplementary Fig. 12). Thus, even in the same rotary state, the reduced and oxidized forms do not have the same spatial arrangements between the subunit components. Moreover, the reduced γ-ε central shaft is slightly further away from the top of the *c*_14_ ring than the oxidized form (rotary state 1: 1.75 Å; rotary state 2: 2.55 Å; rotary state 3: 1.02 Å) (Fig. 4c). Rather than resulting from one single structural change, these concerted movements may synergistically contribute to the differences in efficiency, resulting in a change in the rotation transmission from the membrane rotor to the central shaft in the reduced and oxidized enzymes. Thus, the conformational changes between different redox γ subunits are likely to collaborate with other subunits to influence the rotation of the ATP synthase motor. However, because the movements between these subunits are small, these findings will need further investigation on higher resolution structures to define the trajectory of the movements. Due to its highly flexible nature, this may require further technical development to derive high-resolution structures in the future.

## Discussion

What makes the chloroplast ATP synthase unique from other ATP synthases is the ability to modulate its enzymatic activity through different redox states^6,52^. In this work, we performed a redox titration on the spinach CF_1_F_O_ enzyme, investigating the structures of different CF_1_F_O_ redox forms using single-particle cryo-EM. Single-particle cryo-EM enables direct imaging of the molecular motors, and the subsequent image classification allows the data to be sorted into different conformations. Our cryo-EM reconstructions showed a complete view of the enzyme complexes, composed of α_3_β_3_δγε*abb*′*c*_14_ subunits (Fig. 1c), in different redox and rotary states. The overall architecture, especially the oxidized form, was consistent with the previous CF_1_F_O_ structural data^18^. The resolutions of our 3D densities were anisotropic, which implies that either multiple metastable states co-exist or the enzyme features structural differences in the mobility of domains.

Previous cryo-EM studies and our structures of the ATP synthase showed different proportions of the three rotary states: rotary state 1 is the most populated, and rotary state 3 is the least populated^18,42,53^. This uneven proportion of the particle distribution implies that different energy levels are populated in individual rotary states while the motor rotates. As such, in the oxidized and control samples, the rotary state 1 is more energetically stable than the rotary state 3. While the disulfide bond was reduced, the proportions of the rotary state 2 and 3 were increased and that of the rotary state 1 was decreased (Supplementary Figs. 2b, 3b). Assuming that the proportion of the rotary state follows the Boltzmann distribution, our analysis may implicate that the energy differences for the transitions between the rotary states of the reduced form are lower than those of the oxidized form, allowing a smooth transition in the rotary action and thereby a fast rotation. On the other hand, single-molecule measurement of the CF_1_F_O_ showed that the oxidized rotor can frequently take long pauses during rotation^54^. The oxidized state is very likely to stabilize certain rotary states, leading to an uneven rotation. The stabilization may result from the interaction network of the β hairpin structures of the γ subunit with its neighbor motifs.

Superposition of the reduced and oxidized γ subunit showed that the two hairpin structures may be the key to modulating the ATP synthase activity (Fig. 3b). The torsional restraints given by the disulfide linkage may stabilize the structure, strengthening the interactions between the γ and β subunits. When the γ subunit is reduced, the breakdown of the disulfide bond and instability of this short-hairpin loop probably confers an entropic gain to relax the mechanical torsion. On the other hand, the concerted movements of the CF_1_F_O_ caused by the γ redox change modulate the properties of the transmission between the rotors. The γ subunit may act as a molecular clutch to control the rotation of the CF_1_F_O_ motor. Understanding the mechanism in more detail will be required for further confirmation from biochemical studies or high-resolution structures.

**Fig. 5:**
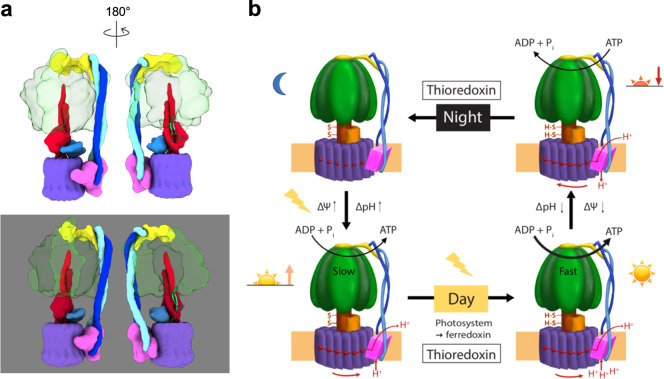
Proposed working model for the light regulatory mechanism. **a** Cartoon schematics of the redox modulation. Upper and lower models are the reduced and oxidized states, respectively. Color codes are the same as in Fig. 1c and the β hairpin structures of the γ subunit are shown in light green. The two redox states are aligned in the same view. **b** At night, no energy input from light is available for the photosynthetic electron transport chain, and thus, no electrochemical potential (ΔΨ) and proton gradient (ΔpH) are generated. The oxidized γ subunit prevents CF_1_F_O_ from hydrolyzing ATP. During the day, light induces charge separation to generate an electrochemical potential across the membrane. Although the CF_1_F_O_ begins to synthesize ATP molecules, the γ subunit is still oxidized while ΔΨ is small. The rate of ATP synthesis is not at its maximum. At sunrise, thioredoxin subsequently reduces the γ subunit, fully activating CF_1_F_O_. The molecular motor, consisting of the γ-ε central shaft and the *c*_14_-ring, is free to rotate at full speed to maximize its ATP synthesis activity. Three ATP molecules per rotation of the *c*_14_ ring are produced. At sunset, the membrane becomes de-energized, leading to small ΔΨ and ΔpH, and the ATP hydrolysis starts to take place. To prevent ATP loss from excess ATP hydrolysis, the γ subunit is then oxidized again. This process of light regulation and redox modulation on the CF_1_F_O_ will cyclize daily.

From our results summarized above, a mechanistic view of the molecular redox modulation of CF_1_F_O_ can be deduced. When the plant leaves are in the darkness, the γ subunit of the CF_1_F_O_ is oxidized to form a disulfide linkage, which introduces a torsional constraint on the structures of β hairpins 1 and 2. The hairpin structures also stabilize the interactions between the βDELSEED motif and the coiled coil of the γ subunit. In addition, the membrane-embedded subunit *a* and membrane *c* ring closely interact with the central shaft. These may limit the rotary actions to prevent unnecessary ATP hydrolysis and minimize the loss of ATP in the darkness (Fig. 5b). At sunrise, when the photosynthetic electron transport chain of the plant is activated, PSI transfers the electron to ferredoxin, which reduces thioredoxin and subsequently reduces the γ subunit of CF_1_F_O_ (Fig. 5). The disconnected cysteines alleviate the torsion on the β hairpin 1 and remove the constraints of the β hairpin 2 of the γ subunit. This leads to multiple concerted movements within the CF_1_F_O_ assembly to transition into efficient ATP synthesis. At the same time, the electron transport from the photosystem generates the proton gradient across the thylakoid membranes, which drives the *c*-ring rotation with less friction and synthesizes the ATP molecules. These synergistic actions would then keep the ATP synthesis in the activated state at full speed during the day.

In summary, we determined the first structure of the active, reduced chloroplast ATP synthase to our knowledge. Our cryo-EM structures of the chloroplast ATP synthase revealed significant differences of the reduced and oxidized enzyme triggered by distinct redox states of the γ subunit. These results provide a fundamental framework of how CF_1_F_O_ is regulated by the presence or absence of light. Our first structure of the active, reduced CF_1_F_O_ brings together the experimental observations with the lines of structural evidence of the redox modulation that was investigated over multiple decades from researchers in the field into one consistent picture. This reveals the structural basis for light-dependent regulation of ATP synthesis in chloroplasts as a robust model for redox-dependent molecular switching.

## Methods

### Purification of the chloroplast ATP synthase complex

Samples of the chloroplast ATP synthase complexes were purified from baby spinach, *Spinacia oleracea*, as previously described for *Heliobacterium modesticaldum*^55^. Five kilograms of fresh baby spinach leaves (Fresh Express, Chiquita Brands International Inc.) were stored at 4 °C in the darkness for at least 3 days. Selected leaf tissues were placed in ice-cold water and subsequently homogenized in 100 mM Tricine (pH 8.0), 400 mM sucrose, and 2 mM MgCl_2_. The tissue lysate was centrifuged at 16,000 × *g* at 4 °C for 25 min.

Cell lysis was performed at 4 °C in the darkness using osmotic shock, followed by centrifugation at 16,000 × *g* for 30 min at 4 °C. The resuspended pellet was homogenized to reach a final concentration of 5 mM chlorophyll in 50 mM Tricine (pH 8.0), 400 mM sucrose, and 4 mM MgCl_2_. The membrane part was then solubilized in a buffer containing 10 mM Tricine-NaOH (pH 8.0), 100 mM sucrose, 2.5 mM MgCl_2_, 2.5 mM KCl, 2 mM ATP, 10% (w/v) NH_4_SO_4_, 50 mM dithiothreitol (DTT), 12.5 mM sodium cholate, and 30 mM β-D-octylglucoside (OG; Glycon Biochemicals, Luckenwalde, Germany). The supernatant was collected using centrifugation at 208,000 × *g* for 60 min at 4 °C. The purified complexes were precipitated using ammonium sulfate at the cut-off saturated concentrations of 32.5 to 45% (v/v). The precipitates were collected by centrifugation at 12,000 × *g* for 15 min at 4 °C and resuspended in 30 mM NaH_2_PO_4_ (pH 7.2), 200 mM sucrose, 2 mM MgCl_2_, 0.5 mM EDTA, and 4 mM n-dodecyl-β-D-maltoside (β-DDM; Glycon Biochemicals, Luckenwalde, Germany). The protein samples were further purified by discontinuous sucrose gradient centrifugation with the steps of 20, 28, 36, 44, 52, and 60% (w/v) sucrose in the gradient buffer consisting of 30 mM NaH_2_PO_4_ (pH 7.2), 2 mM MgCl_2_, 0.5 mM EDTA, 1 mg/ml asolectin, and 8 mM β-DDM. The bands at the interface between 44 and 52% sucrose interface were collected and loaded onto a POROS 20 HQ anion-exchange column (Thermo Fisher Scientific/Life Technologies, Waltham, MA) in 10 mM Tris-Cl (pH 8.0), 10 mM MgCl_2_, and 0.4 mM β-DDM with an elution gradient generated by 500 mM NaCl. The collected peak fraction was further purified using size-exclusion chromatography (Superdex 200 10/300 column, GE Healthcare, Chicago, IL) with 10 mM Tris-Cl (pH 8.0), 2 mM MgCl_2_, 500 µM ATP, and 0.4 mM β-DDM. The peak fraction was characterized using SDS-PAGE electrophoresis with silver staining and negative-stain electron microscopy (EM).

### Luciferin/luciferase ATP synthesis assay

Functional assays were followed by the previous methods with modifications^23,56^. Liposomes were prepared by mixing L-α-phosphatidylcholine (Egg PC, Avanti) and L-α-phosphatidic acid (Egg PA, Avanti) at a lipid-to-protein ratio (LPR) of 19:1 (w/w) in 10 mM Tricine (pH 8.0), 100 μM EDTA, 500 µM DTT, 7.2 mg/ml sodium cholate, and 3.6 mg/ml sodium desoxycholate. The mixture was sonicated to form an emulsion and dialyzed at 30 °C for 5 h against 15 L of 10 mM Tricine (pH 8.0), 0.2 mM EDTA, 0.25 mM DTT, and 2.5 mM MgCl_2_. The purified proteins were reconstituted into liposomes by detergent removal with Bio-Beads (Bio-Rad SM_2_). The pH gradient was generated using a pH-pump step method^23,56^. Valinomycin was used to generate the potassium gradient across the membrane, leading to an additional chemiosmotic potential, Δφ, across the membrane when the buffers of high (pH 4.7) and low (pH 8.7) proton concentrations were mixed. The luminescence generated with luciferin and luciferase was detected using an LKB WALLAC-1250 luminometer (Vienna, Austria).

### Electron microscopy of the enzyme complex

The purified complexes were negatively stained with 0.75% (w/v) uranyl formate. Electron images were recorded using a Philips CM12 transmission electron microscope (TEM) equipped with a side-mounted CCD camera (Model 791, Gatan, Pleasanton, CA). TEM imaging was operated at an acceleration voltage of 80 keV with the settings of a calibrated magnification of ×18,680, corresponding to a pixel size of 8.03 Å/pixel at the specimen level, and a defocus setting of about −1.6 μm.

For cryo-EM specimen preparation, 5 µl of 10 mg/ml protein sample was applied onto a glow-discharged C-flat holey carbon grid (1.2/1.3–4C or 2/1–4C, Protochips, Morrisville, NC). The grid specimen was plunge-frozen into liquid ethane using a Thermo Fisher/FEI Vitrobot Mark IV (Thermo Fisher/FEI, Hillsborough, OR) with a blotting time of 6 s and at a humidity of 100% at 22 °C. The frozen grid specimens were stored in the liquid-nitrogen dewar before imaging.

The protein samples were mixed with 100 mM DTT or 20 mM iodosobenzoate (IBZ; Sigma-Aldrich, Burlington, MA) to generate the reduced or oxidized samples, respectively. The protein samples were incubated for 10 min before plunge freezing. For the reduced sample, 0.05 mM tentoxin (Cayman Chemical, Ann Arbor, MI) was also added.

The data were collected in the Eyring Materials Center (EMC) at Arizona State University (ASU) and the European Synchrotron Radiation Facility (ESRF)^57^. The samples were imaged at the EMC using a Thermo Fisher/FEI Titan Krios TEM (Thermo Fisher/FEI, Hillsborough, OR) at an accelerating voltage of 300 kV, and the dose-fragmented frames were recorded on a Gatan K2 Summit direct electron detector (DED) camera in super-resolution mode (Gatan, Pleasanton, CA). The imaging was performed in the nanoprobe mode with a C2 aperture diameter of 50 µm. The low-dose procedure was applied with a defocus setting ranging from −1.5 to −4.0 µm and a nominal magnification of ×48,077, which corresponds to a physical pixel size of 1.04 Å/pixel at the specimen level. The beam intensity was adjusted to a counting rate of 2 counts/sub-pixel/s on the camera. The exposure time was 6 s with a subframe rate of 200 ms for each movie, accumulating to a total dosage of 43.5 e^−^/Å^2^. For the images of oxidized sample, the beam-image shift was applied to promote the data acquisition speed^58^. The data collection was automated using SerialEM program (version 3.7) with customized macros^59^.

The electron movies collected at the ESRF were recorded on a Thermo Fisher/FEI Titan Krios TEM equipped with a Quantum LS energy filter and a Gatan K2 Summit DED camera. The image data was recorded in the counting mode at a nominal magnification of 130,000 X, corresponding to a pixel size of 1.053 Å/pixel at the specimen level. The defocus range was set from −1.0 to −2.8 µm. The exposure time was 7 s for each movie, accumulating to a total dosage of 49 e^−^/Å^2^. The beam-image shift was applied during data collection for multiple images per foil-hole. The data collection was automated using EPU software (version 1.11; Thermo Fisher/FEI, Hillsborough, OR).

### Image processing

3935, 2064, and 1637 movies were collected for the oxidized, reduced, and control samples, respectively. For the dataset of the oxidized and control samples, the IMOD program (version 4.9) “clip” was used to unpack and gain-normalize the movie data^60^. The movie frames were motion corrected and dose weighted using the MotionCor2 program (version 1.2.1)^61^, and the final frame average was performed with a Fourier cropping at the spatial frequency of 2 and 1.5 times physical Nyquist frequency, resulting in a pixel size of 1.04 and 0.788 Å/pixel for the movies of the oxidized and control samples, respectively. The processing workflow was generally followed the RELION program (version 3.1-beta-commit-da823c)^62^. The defocus and astigmatism of individual image was estimated by CTFFIND4 (version 4.1.13)^63^. The particle images were automatically selected using a template-based approach that was implemented in Relion software. The false positives were removed by iterative two-dimensional (2D) classification procedures and selection of 2D class averages. The final particle numbers used for 3D reconstruction and classification were 552,893, 108,691, and 208,371 for oxidized, reduced, and control samples, respectively.

The 3D initial densities were built using stochastic gradient descent method that was implemented in Relion software, except the initial density for the control sample was built using “sxviper.py” (SPARX) supplied with 2D class averages^64^. The initial densities were then refined against the particle images using regularized likelihood optimization in Relion. The density maps of different states and samples were generated by iterative 3D classification and refinement. To further improve the densities of the F_1_ region, the signal subtraction and focused refinement was applied^65^. The final density map was sharpened by an estimated *b* factor using Guinier plot^66^ and the modulation transfer function of the DED camera at 300 keV. The gold-standard Fourier-shell correlation (FSC) was used to estimate the overall resolution of the density map^67^. Local resolution was estimated using Relion software.

### Modeling

The initial templates used for modeling were the structures of the spinach chloroplast ATP synthase (PDB codes: 6FKF, 6FKH, and 6FKI)^18^. The initial rigid-body fitting of the templates was performed against one of the half maps using “Fit in the Volume” function implemented in the UCSF Chimera (version 1.14)^68^. The density maps that were not sufficient to identify the side chains were only used to model the coordinates of the main chain. The atomic coordinates were rebuilt and fit to the density map using Coot (version 0.9-pre). The residue assignment was based upon the densities of bulky side chains, such as aromatic residues. The ligand configuration of the tentoxin coordinate was optimized using AM1 (Austin Model 1)^69^ quantum-mechanical method in eLBOW program^70^. The rebuilt model was refined against the density map using “phenix.real_space_refine” program in the PHENIX suite (version: 1.16–3546 or 1.17.1–3660)^71^. The representation of the atomic models was made by the UCSF Chimera or ChimeraX (version 0.91)^68,72^.

### Statistics and reproducibility

Individual cryo-EM density maps are representative from over 10,000 particle images and the observations were described by quantitative data (Supplementary Figs. 2, 3, 4). The resolution of the reconstruction was estimated by randomly dividing dataset into two independent groups and determined using the gold-standard FSC method^67^.

### Reporting summary

Further information on research design is available in the Nature Research Reporting Summary linked to this article.

## Supplementary information

Supplementary Information

Description of Additional Supplementary Files

Supplementary Data 1

Reporting Summary

## Data Availability

Cryo-EM density maps (MRC format) were deposited in the Electron Microscopy Data Bank (EMDB) under accession numbers EMD-21270 (R1), EMD-21271 (R1-F1), EMD-21268 (R2), EMD-21269 (R2-F1), EMD-21266 (R3), EMD-21267 (R3-F1), EMD-21264 (O1), EMD-21265 (O1-F1), EMD-21262 (O2), EMD-21263 (O2-F1), EMD-21241 (O3), EMD-21239 (C1), EMD-21240 (C1-F1), EMD-21238 (C2), and EMD-21235 (C3). Model coordinates were deposited in the Worldwide Protein Data Bank (wwPDB) under accession numbers 6VON (R1), 6VOO (R1-F1), 6VOL (R2), 6VOM (R2-F1), 6VOJ (R3), 6VOK (R3-F1), 6VOH (O1), 6VOI (O1-F1), 6VOF (O2), 6VOG (O2-F1), 6VMG (O3), 6VMB (C1), 6VMD (C1-F1), 6VM4 (C2), and 6VM1 (C3). All the data are available in the EMDB and wwPDB database or from the corresponding author upon request. Data of the luminescence measurements for the reconstituted CF_1_F_O_ function in different redox states are presented in Supplementary Data 1.
