# Supplementary material for: Structural basis of redox modulation on chloroplast ATP synthase
Source: Commun Biol. 2020 Sep 2;3:482. doi: 10.1038/s42003-020-01221-8 (PMC7468127; doi:10.1038/s42003-020-01221-8)
Supplement: Supplementary file 1 — Supplementary Information [file 42003_2020_1221_MOESM1_ESM.pdf]

SUPPLEMENTARY FIGURES

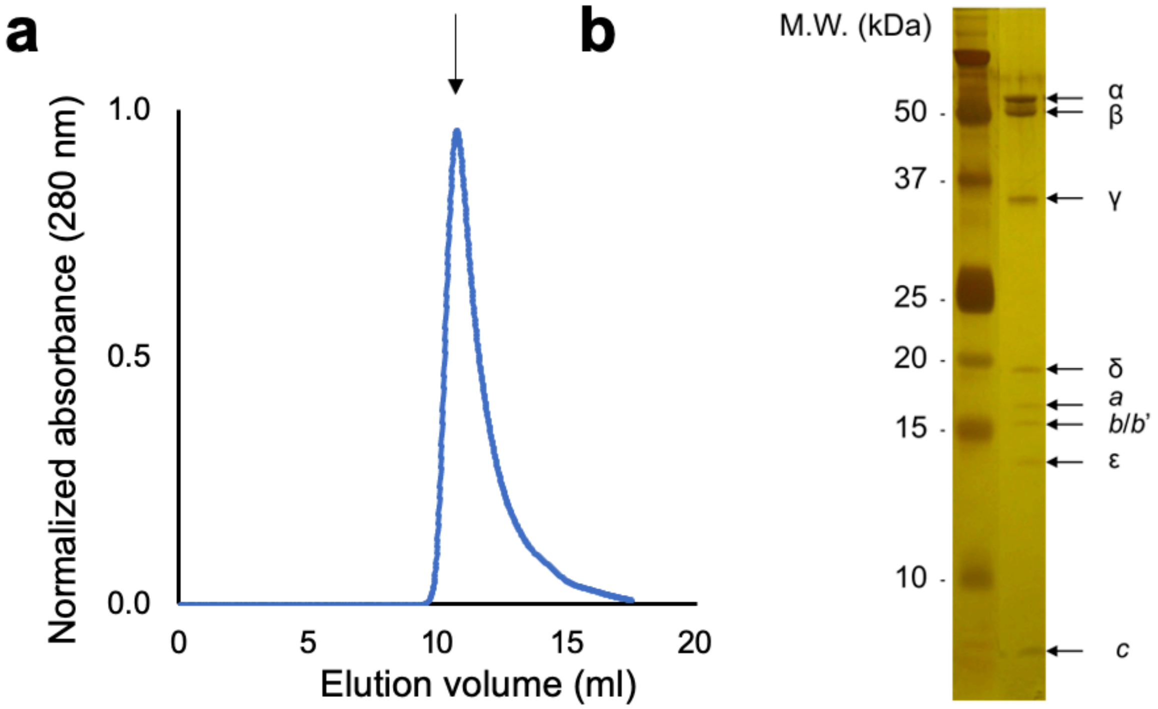

Supplementary Figure 1 | Characterization of the isolated spinach chloroplast ATP synthase complex. a, Size-exclusion chromatogram of the purified complex. The peak fraction was collected as the arrow indicated for the intact CF_1F_O. **b,** Silver staining of an SDS-PAGE gel of the isolated protein complex. Gel bands of individual subunits of the enzyme complex are labeled based on their molecular weights.

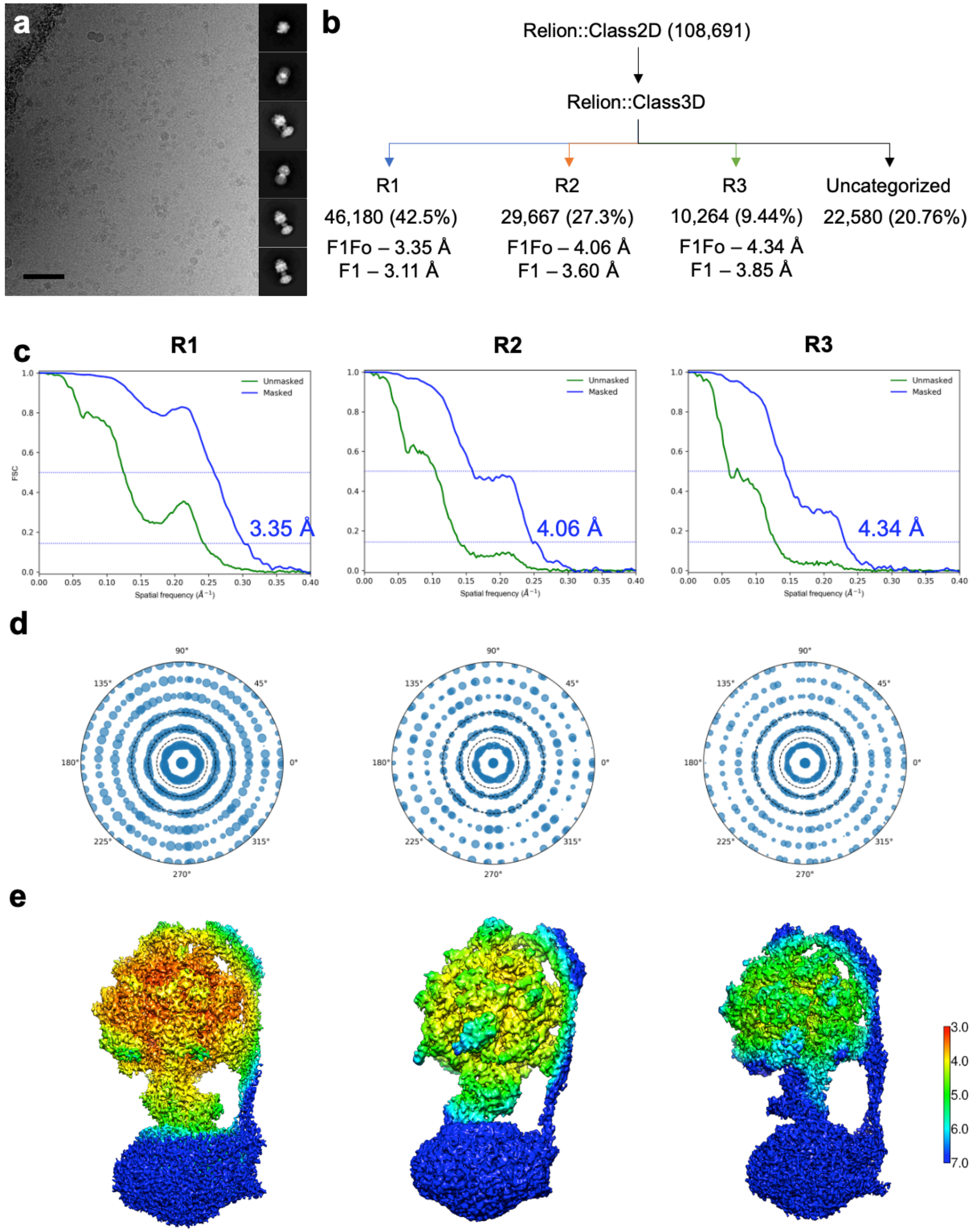

Supplementary Figure 2 | Single-particle cryo-EM of the reduced chloroplast ATP synthase complex (CF_1F_O). a, Electron micrograph of cryogenic CF_1F_O. Proteins are

in black contrast on white background. The particles represent different orientations of the enzyme with a size of about 12 nm along the longest axis and 8 nm at the shortest axis. Scale bar indicates 50 nm. **b,** Schematics of the image classification. The reduced and rotary state 1 is abbreviated as R1, and the same naming convention is for R2 and R3.   **c,** Golden Fourier shell correlation (FSC) plot against spatial frequencies. Resolution values reported are for FSC=0.143. **d,** Distribution of the azimuthal and tilt angles for the 3D reconstruction. Tilt angle is in the radial direction. **e,** Local resolution estimation. Color bar indicates the resolution ($Å^{-1}$).

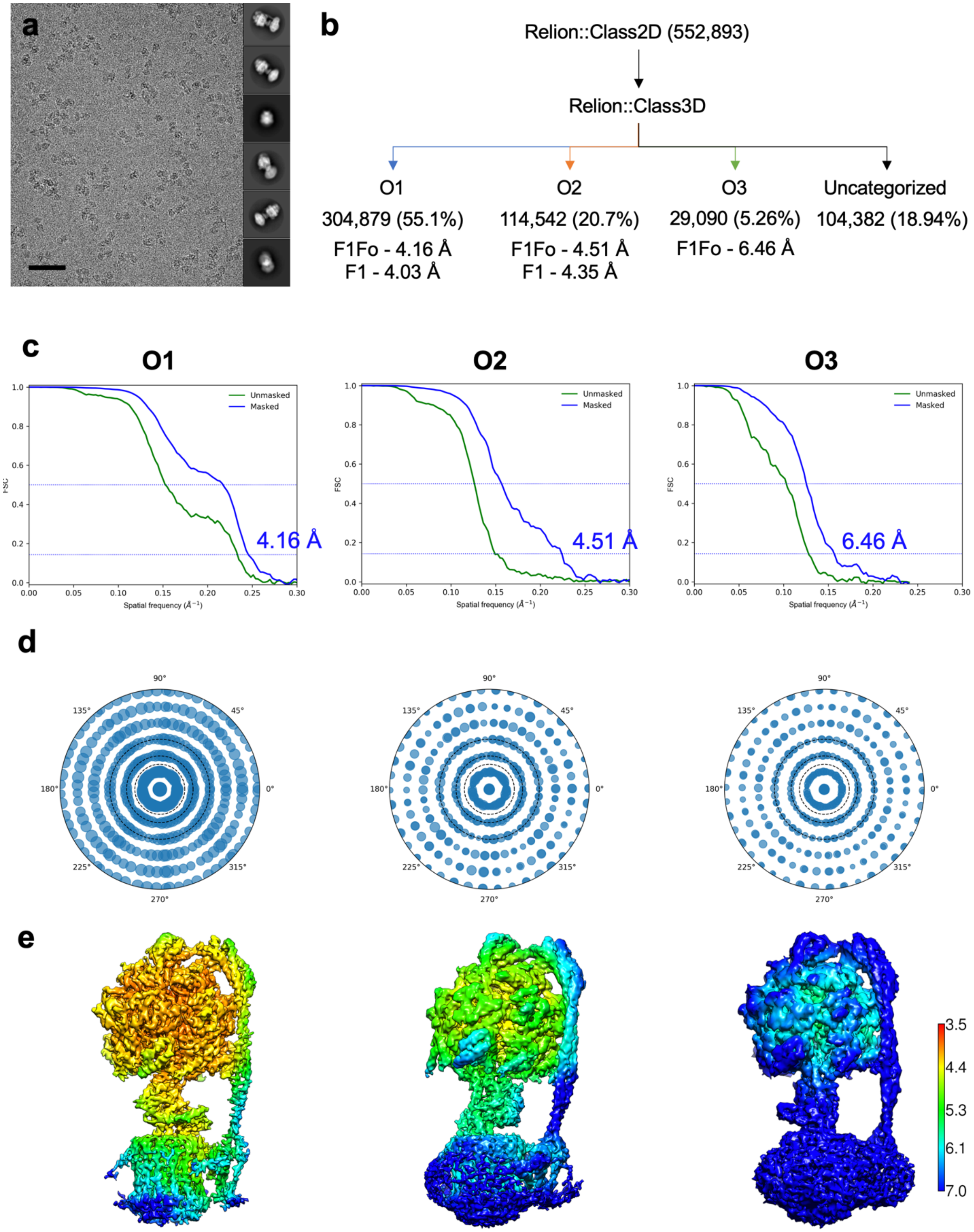

Supplementary Figure 3 | Single-particle cryo-EM of the oxidized chloroplast ATP synthase complex (CF_1F_O). a, Electron micrograph of cryogenic CF_1F_O. Proteins are

in black contrast on white background. The purified complex is shown in black contrast. Scale bar indicates 50 nm. **b,** Schematics of the image classification. The oxidized and rotary state 1 is abbreviated as O1, and the same naming convention is for O2 and O3. **c,** Golden Fourier shell correlation (FSC) plot against spatial frequencies. Resolution values reported are for FSC=0.143. **d,** Distribution of the azimuthal and tilt angles for the 3D reconstruction. Tilt angle is in the radial direction. **e,** Local resolution estimation. Color bar indicates the resolution ($Å^{-1}$).

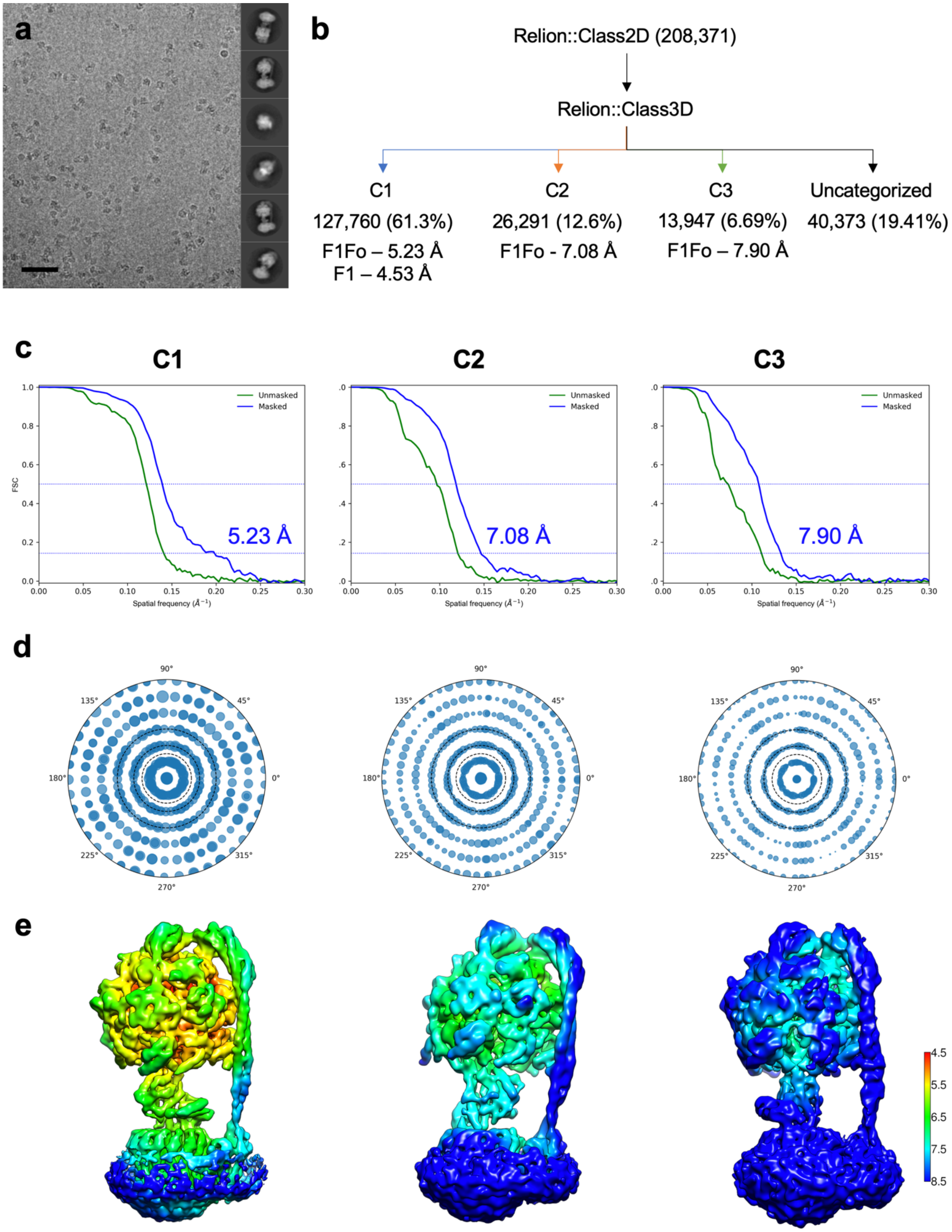

Supplementary Figure 4 | Single-particle cryo-EM of the control chloroplast ATP synthase complex (CF_1F_O). a, Electron micrograph of cryogenic CF_1F_O. Proteins are

in black contrast on white background. The purified complex is shown in black contrast. Scale bar indicates 50 nm. **b,** Schematics of the image classification. The control and rotary state 1 is abbreviated as C1, and the same naming convention is for C2 and C3. **c,** Golden Fourier shell correlation (FSC) plot against spatial frequencies. Resolution values reported are for FSC=0.143. **d,** Distribution of the azimuthal and tilt angles for the 3D reconstruction. Tilt angle is in the radial direction. **e,** Local resolution estimation. Color bar indicates the resolution ($Å^{-1}$).

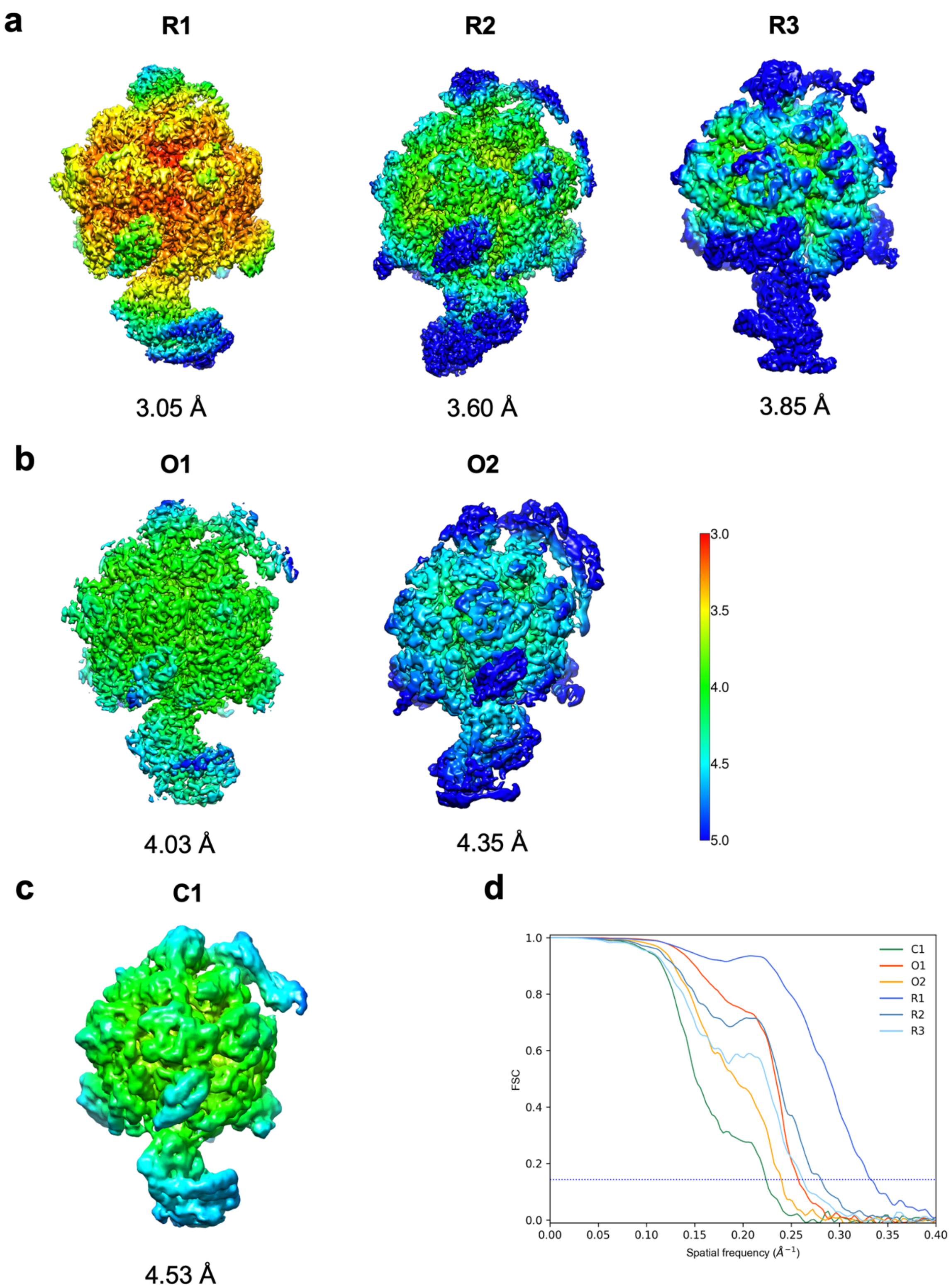

Supplementary Figure 5 | Cryo-EM density maps of the F_1 domains. The 3D densities of the F_1 domains were refined against the 2D particle images with the

densities of the F_O domain subtracted. Color coding represents the estimated local resolution in $Å^{-1}$. **a,** Reconstruction of the F_1 domains of the R1, R2, and R3 states. **b,** Reconstruction of the F_1 domains of the O1 and O2 states. **c,** Reconstruction of the F_1 domains of the C1 state. **d,** Fourier shell correlation (FSC) plot versus spatial frequencies of individually focused refinement. Blue dash line indicates the FSC=0.143.

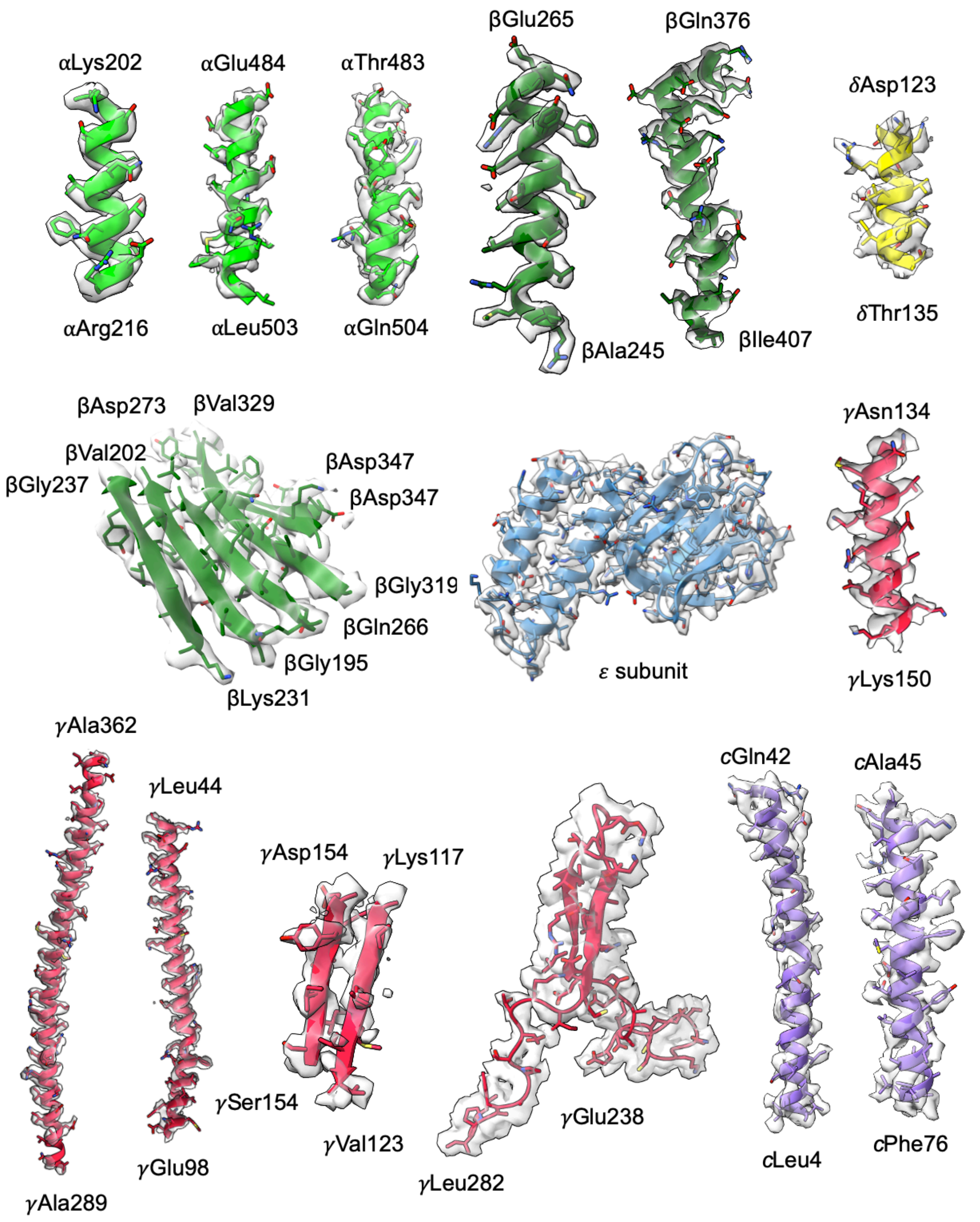

Supplementary Figure 6 | Atomic modeling of the cryo-EM density of the reduced CF_1F_O. Cryo-EM densities are shown in grey surfaces. Color codes are the same as used in **Fig. 1c**.

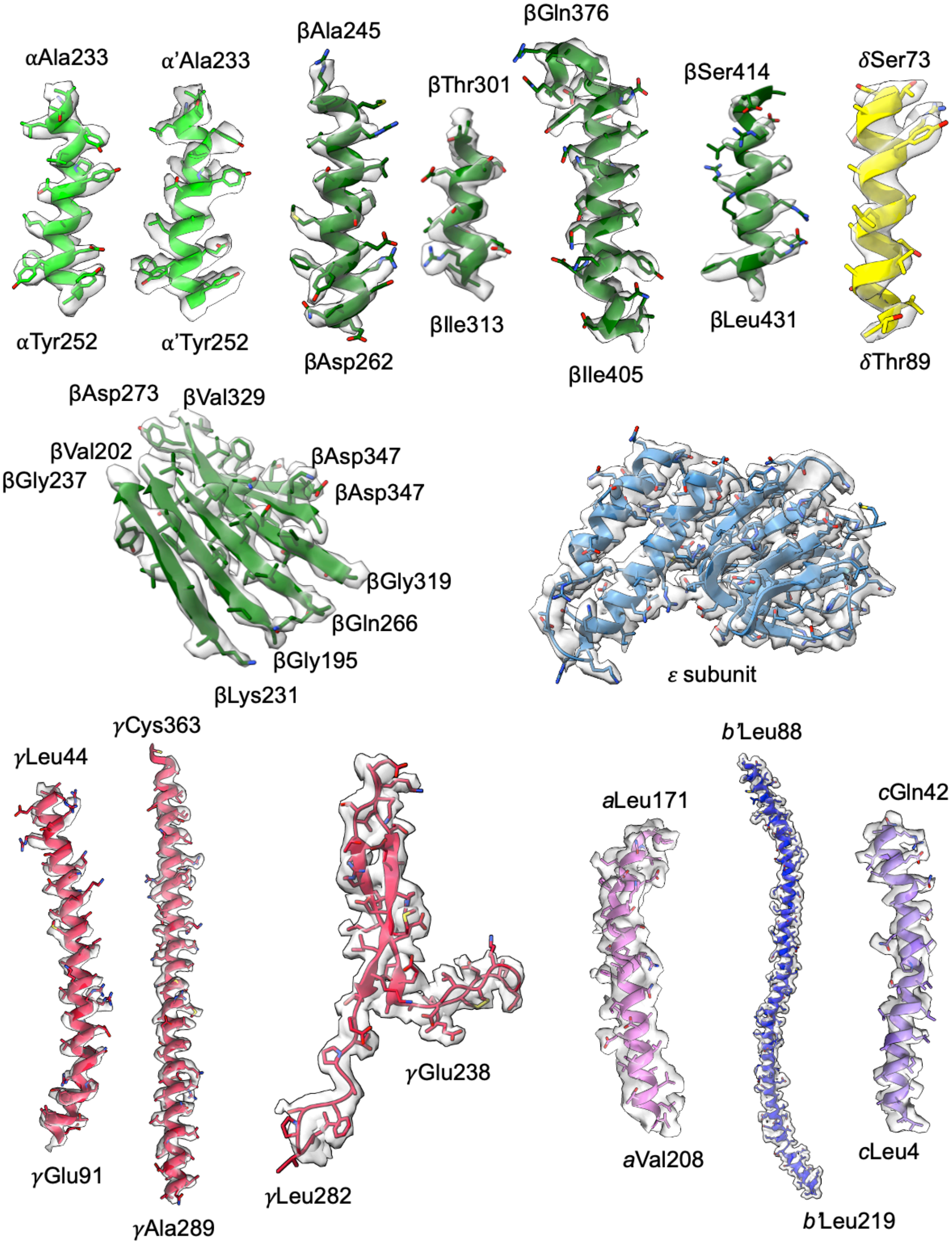

Supplementary Figure 7 | Atomic modeling of the cryo-EM density of the oxidized CF_1F_O. Cryo-EM densities are shown in grey surfaces. Color codes are the same as used in **Fig. 1c**.

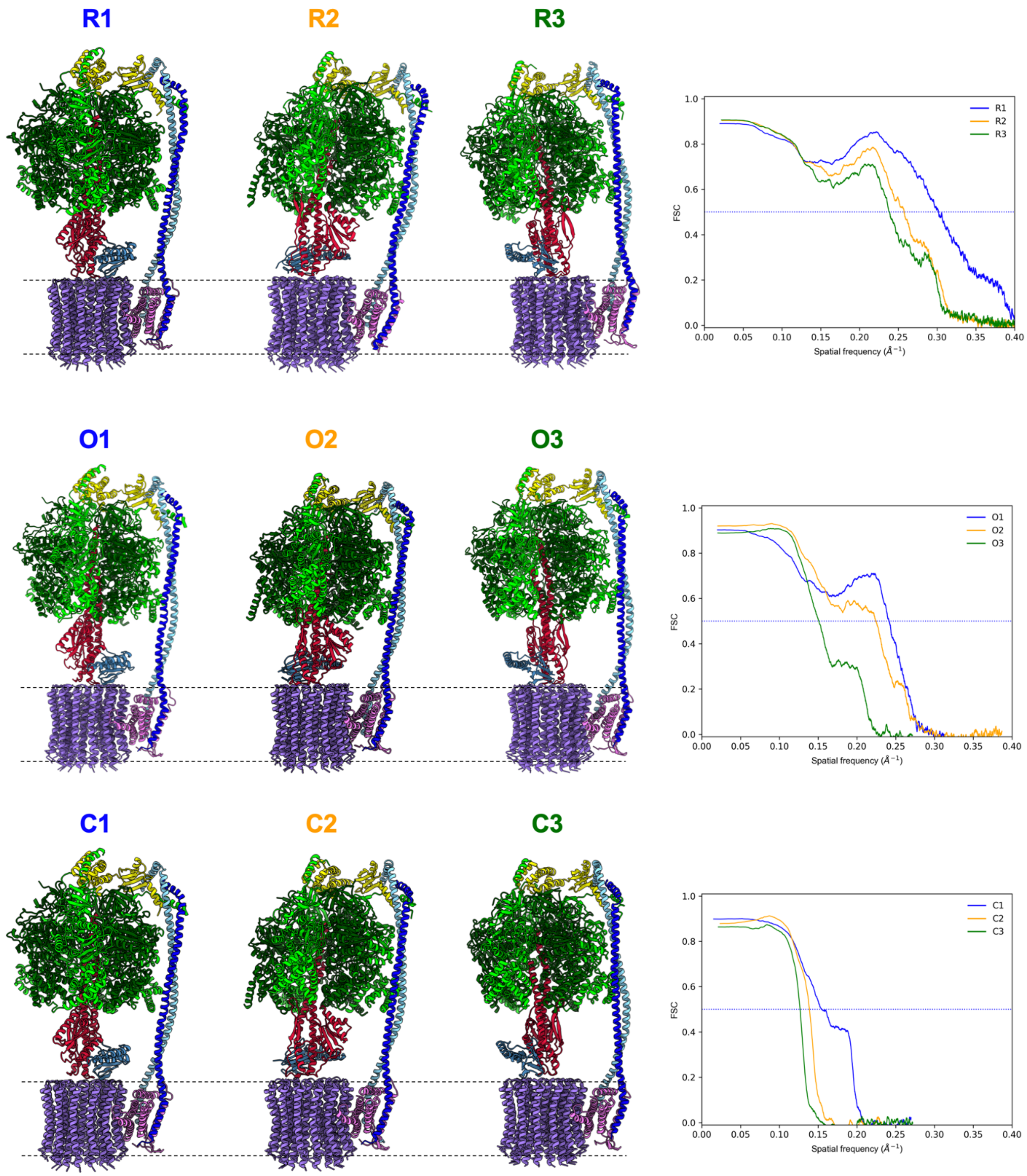

Supplementary Figure 8 | Atomic models of individual rotary and redox states of the CF_1F_O. Color codes of the structural models are the same as used in **Fig. 1c**. Right panel are the Fourier shell correlation (FSC) plots against spatial frequencies (model versus cryo-EM density map). Blue dash line indicates FSC=0.5.

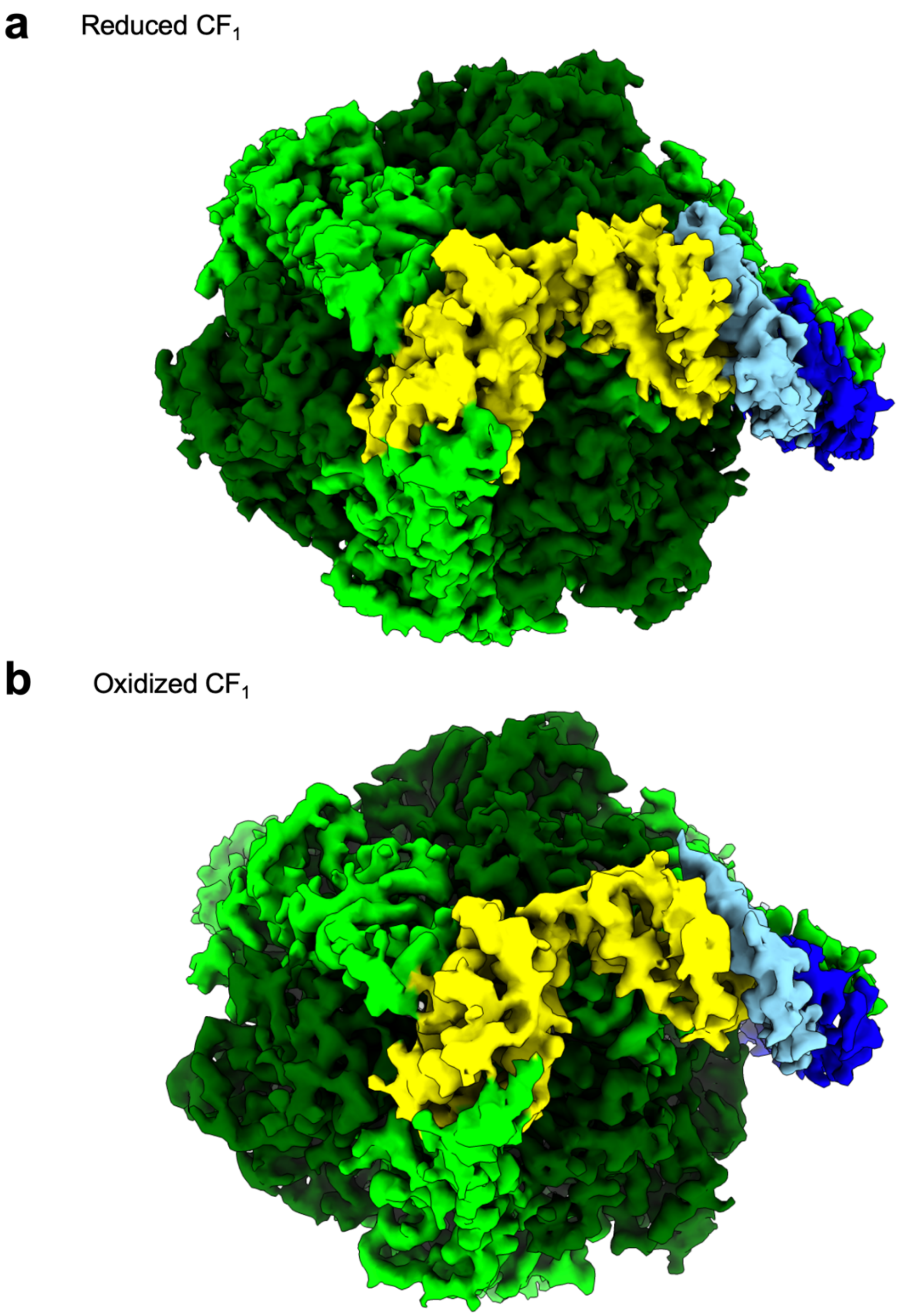

Supplementary Figure 9 | Top view of the cryo-EM densities of the CF_1F_O in reduced and oxidized states. Spatial organizations of the catalytic unit and δ and peripheral stators are similar in the reduced (**a**) and oxidized states (**b**). Color codes: δ subunit (yellow), α subunits (green), β subunits (dark green), and peripheral stator *bb'* (blue and light blue).

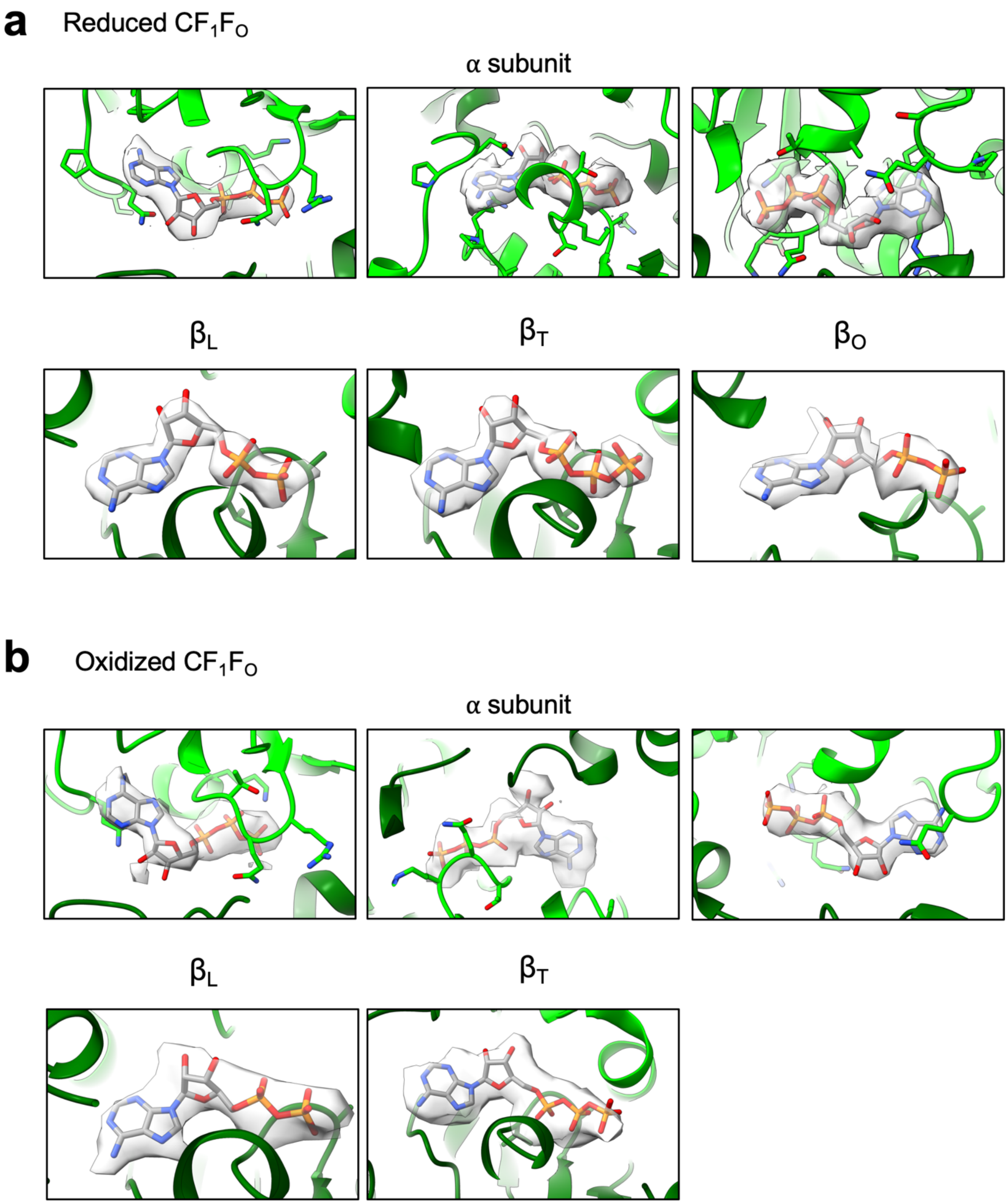

Supplementary Figure 10 | Nucleotide-binding sites of the reduced and oxidized forms of the CF_1F_O. a, Nucleotide-binding sites of the reduced CF_1F_O. β_L, β_T, and β_O represent the loose (ADP), tight (ATP), and open sites for the nucleotide binding, respectively. Cryo-EM densities are in grey surfaces. Light green and dark green are

for the α and β subunit, respectively. Proteins are in cartoon representation and nucleotides in stick representation. **b,** Nucleotide-binding sites of the oxidized CF_1F_O.

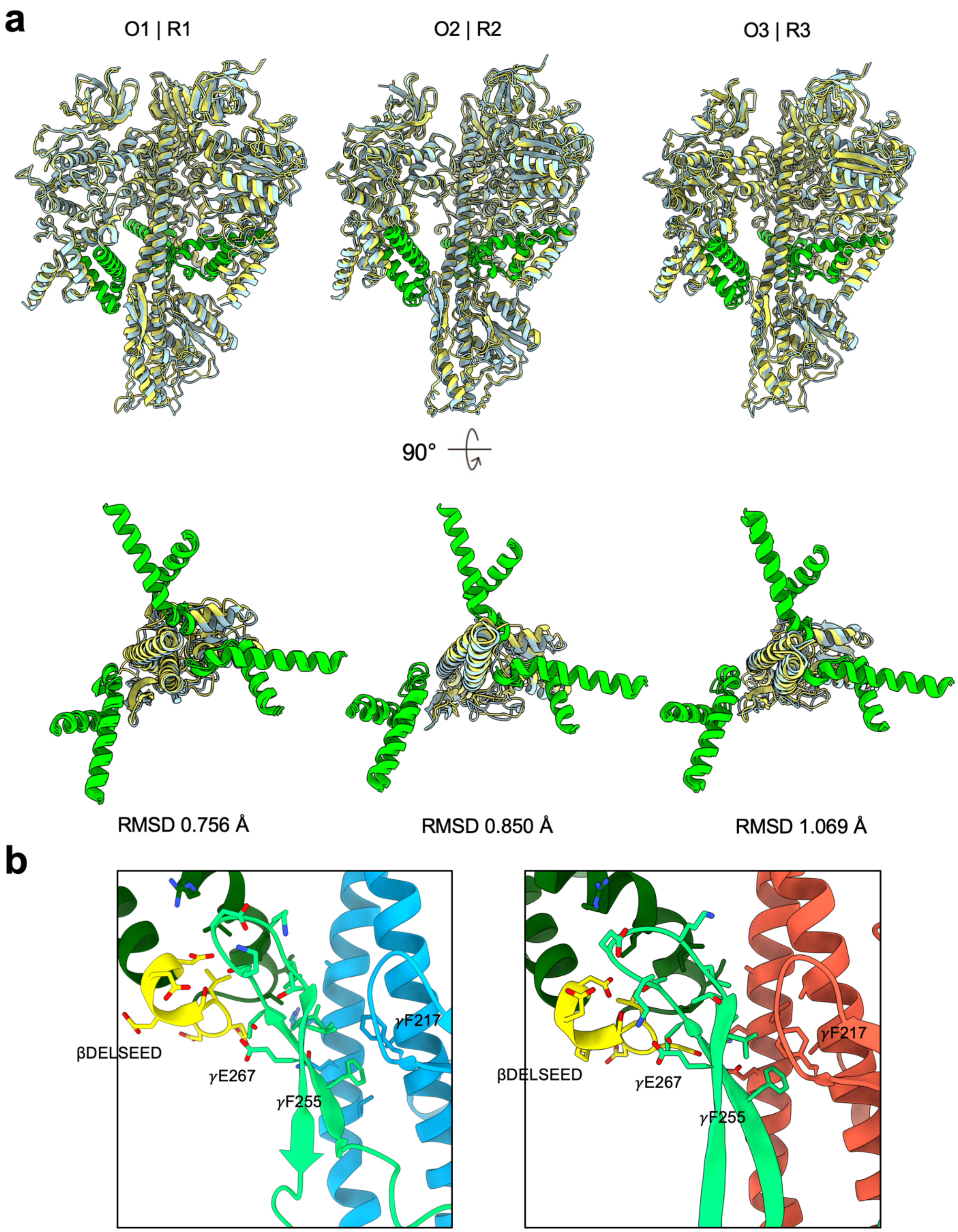

Supplementary Figure 11 | Interactions between the βDELSEED motif and *γ* subunit in the reduced and oxidized CF_1F_O. a, *γ* and β subunits of the CF_1F_O in

reduced and oxidized states are superimposed in individual rotary states. Light blue and yellow are for reduced and oxidized form, respectively. βDELSEED motifs are in green. RMSDs were calculated for the superposition of the βDELSEED motifs and γ subunit between the two redox states. **b,** Interactions between the β hairpin 2 of the γ subunit (green) and βDELSEED motif (yellow). Dark green is the β subunit. Light blue and orange are for reduced and oxidized γ subunit, respectively.

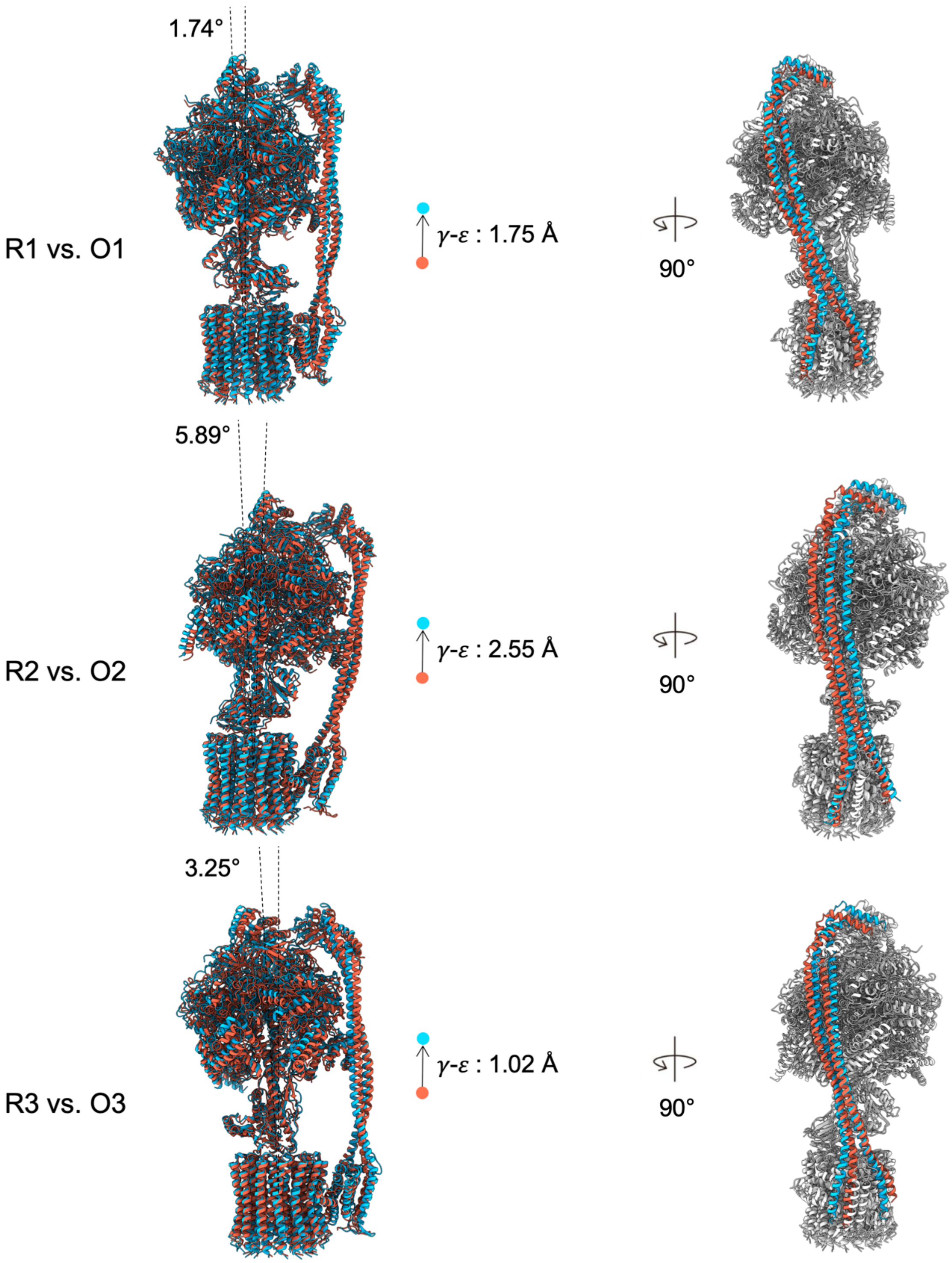

Supplementary Figure 12 | Structure superpositions of the CF_1F_O full complexes of the reduced and oxidized states in individual rotary states. Superpositions of the reduced (light blue) and oxidized (orange) forms were aligned against the membrane *c* ring, presumably the ring rotor as a rigid body. Using the center of mass of

the *c* ring as a common point, the tilting angles were determined by the extent of movements of the center of mass of the F_1 domain. Translational movement of the γ-ε central shafts was measured using their centers of mass. The right panel shows the movements and conformational changes of the peripheral *bb'* stators.

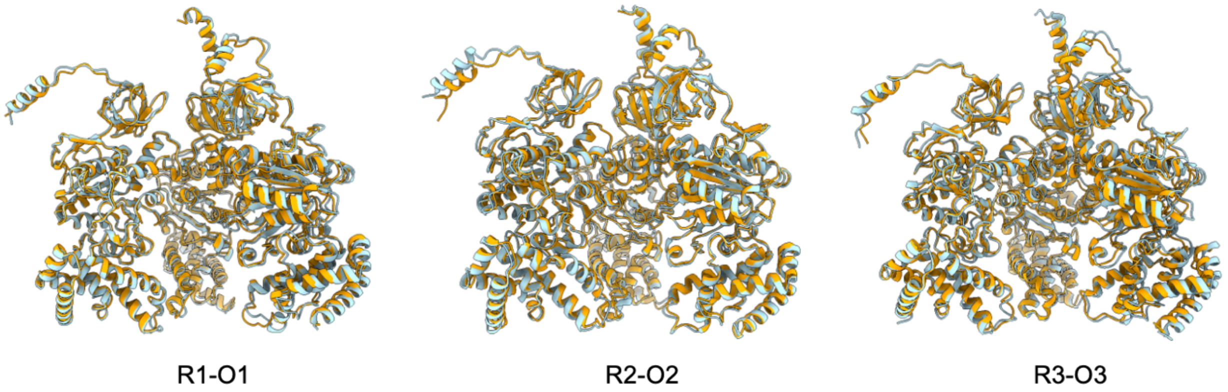

Supplementary Figure 13 | Superpositions of the α subunit in two redox states. Reduced and oxidized forms are light blue and orange, respectively. The RMSDs of the superposition of individual rotary states are 0.768 Å (R1-O1), 0.895 Å (R2-O2), and 1.101 Å (R3-O3). The α subunits of the two redox states does not exert a significant conformational change.
