# Supplementary material for: Structural basis of redox modulation on chloroplast ATP synthase
Source: Commun Biol. 2020 Sep 2;3:482. doi: 10.1038/s42003-020-01221-8 (PMC7468127; doi:10.1038/s42003-020-01221-8)
Supplement: Supplementary file 2 — Description of Additional Supplementary Files [file 42003_2020_1221_MOESM2_ESM.docx]

**Descriptions of Additional Supplementary Files**

**Supplementary Data 1:** Functional measurements of the reconstituted CF1FO in different redox states. The data presents the measured luminescence signals corresponding to the generated ATP molecules.
